# Medical Applications Based on Supramolecular Self-Assembled Materials From Tannic Acid

**DOI:** 10.3389/fchem.2020.583484

**Published:** 2020-10-06

**Authors:** Ruofei Lu, Xiaoqiang Zhang, Xinxiu Cheng, Yagang Zhang, Xingjie Zan, Letao Zhang

**Affiliations:** ^1^Xinjiang Technical Institute of Physics and Chemistry, Chinese Academy of Sciences, Urumqi, China; ^2^University of Chinese Academy of Sciences, Beijing, China; ^3^Department of Chemical and Environmental Engineering, Xinjiang Institute of Engineering, Urumqi, China; ^4^School of Materials and Energy, University of Electronic Science and Technology of China, Chengdu, China

**Keywords:** polyphenol, tannic acid, supramolecular, self-assembled, medical applications

## Abstract

Polyphenol, characterized by various phenolic rings in the chemical structure and an abundance in nature, can be extracted from vegetables, grains, chocolates, fruits, tea, legumes, and seeds, among other sources. Tannic acid (TA), a classical polyphenol with a specific chemical structure, has been widely used in biomedicine because of its outstanding biocompatibility and antibacterial and antioxidant properties. TA has tunable interactions with various materials that are widely distributed in the body, such as proteins, polysaccharides, and glycoproteins, through multimodes including hydrogen bonding, hydrophobic interactions, and charge interactions, assisting TA as important building blocks in the supramolecular self-assembled materials. This review summarizes the recent immense progress in supramolecular self-assembled materials using TA as building blocks to generate different materials such as hydrogels, nanoparticles/microparticles, hollow capsules, and coating films, with enormous potential medical applications including drug delivery, tumor diagnosis and treatment, bone tissue engineering, biofunctional membrane material, and the treatment of certain diseases. Furthermore, we discuss the challenges and developmental prospects of supramolecular self-assembly nanomaterials based on TA.

## Introduction

Supramolecular assembly, which originated from Lehn's groundbreaking work on host–guest self-assembly in 1987 (Lehn, 1988), is a process of spontaneous formation of unique nanostructures by dynamic covalent interactions (Wang et al., 2018a) and non-covalent intermolecular interactions (Caulder and Raymond, 1999), including hydrogen bonds, hydrophobic interactions, electrostatic interactions, van der Waals forces, and π-π stacking. It originates from biological systems and is widely applied in the biomedical field (Cui and Xu, 2017). Such assemblies are common in nature, such as the supramolecular structure of phospholipids in cellular membranes and actin in eukaryotic cytoplasm (Kim et al., 2019). In biological systems, supramolecular assembly involves a variety of functions, such as environmental demarcation, molecular transport and release, and cell–extracellular interactions and communication (Ariga et al., 2019). Inspired by the natural supramolecular assembly, scientists have developed a variety of supramolecular self-assembled biomaterials, including hydrogels, nanoparticles/microparticles, hollow capsules, and coating films, for the diagnosis and treatment of diseases. Supramolecular materials include monodisperse molecules (proteins, polysaccharides, and other biomass materials) and complex molecular aggregates (particles, micelles) (Song et al., 2015). Supramolecular assembly can not only produce thermodynamically or dynamically stable nanostructures but also accurately control diagnostic/therapeutic components and stimulus response characteristics to physiological indicators (Wang et al., 2018b), which is very attractive in biology and medicine.

Polyphenols, including tannic acid (TA), (–)-epigallocatechin-3-gallate (EGCG), catechin, lignans, catechol, and pyrogallol, are a large class of plant-derived biocompatible and biodegradable compounds consisting of two or more phenolic units in structure (Wang X. et al., 2018). Because of their unique sources, the most important characteristics of polyphenols are their biological activities, such as anti-inflammatory, anticancer, antibacterial, and antioxidative actions, which have attracted the attention of researchers in the field of biomedicine (Galante et al., 2019; Song et al., 2019). In terms of chemical structure, polyphenol compounds also show rich chemical activity due to their multiple hydrophobic aromatic rings and hydrophilic phenolic hydroxyls groups, which provide abundant reaction sites that can interact with various groups or substances through various non-covalent interactions (Kozlovskaya et al., 2015; Patil et al., 2018) (including hydrogen bonds, hydrophobic interactions, and van der Waals interactions), dynamic covalent binding (Su et al., 2011; Ye et al., 2011; Faure et al., 2013), and metal–organic coordination interactions (Lee et al., 2011; Rahim et al., 2016; Wang Z. et al., 2017). Such prospects serve as the basis for the application of those compounds. In addition, the hydrophilicity of natural polyphenols facilitates the introduction of amphiphilic characteristics into supramolecular systems (Wang et al., 2018b). Through their significant effects on protein signal transduction and expression and cell cycle regulation implicated in the growth, transformation, and metastasis of cells, polyphenols have become a kind of promising multifunctional drug with chemopreventive and anticancer factures capable of inducing apoptosis and inhibiting the growth of cancer cells (Yang et al., 2002). Considering both the excellent structural and functional characteristics offered by natural polyphenols, materials based on polyphenols as structural units have become a hotspot of research. The addition of polyphenols to these materials endows them with some new properties, such as antioxidative, antitumor, antivirus, and bacteriostatic activities, lending to their extensive application potential in food preservation and pharmaceutical production. At present, the strategies employed for supramolecular engineering based on natural polyphenols are not only focused on building various two-dimensional functional metal–phenolic network (MPN) materials, such as capsules or membranes, but also widely used in three-dimensional supramolecular nanomedicines, including disease diagnosis and imaging, as well as drug delivery (Wang et al., 2018b). TA as a kind of polyphenolic organic compound has certain structural properties and biological activity, and it can be used as a building unit for supramolecular assembly to obtain nanomaterials with biological functions. Therefore, TA has attracted the attention of researchers in the field of biomedicine.

Supramolecular self-assembled nanomaterials, especially nanomaterials based on TA, have great potential applications in biomedical fields with respect to, for example, diagnosis, treatment, and medical devices (Zou et al., 2017). In terms of function, TA endows self-assembled nanomaterials with good biocompatibility, antibacterial, antioxidative, and antitumor characteristics; in terms of structure, supramolecular assembly can be driven by a variety of non-covalent or dynamic covalent interactions to regulate the materials on a nanometer scale and provide greater functionality to the assembled materials. The supramolecular assembly of TA combines the biological activity of polyphenols with the structural control advantages of supramolecular assembly to prepare functionalized nanobiomaterials suitable for different applications. Supramolecular materials based on TA with excellent properties, such as stimulus response, self-healing, and memory shaping capabilities, are ideal in biomedical applications (Fan et al., 2017b). Supramolecular assembly of TA can also provide a general and inexpensive platform for the design of multifunctional films (Wu et al., 2015a; Dong et al., 2017).

**Figure 1:**
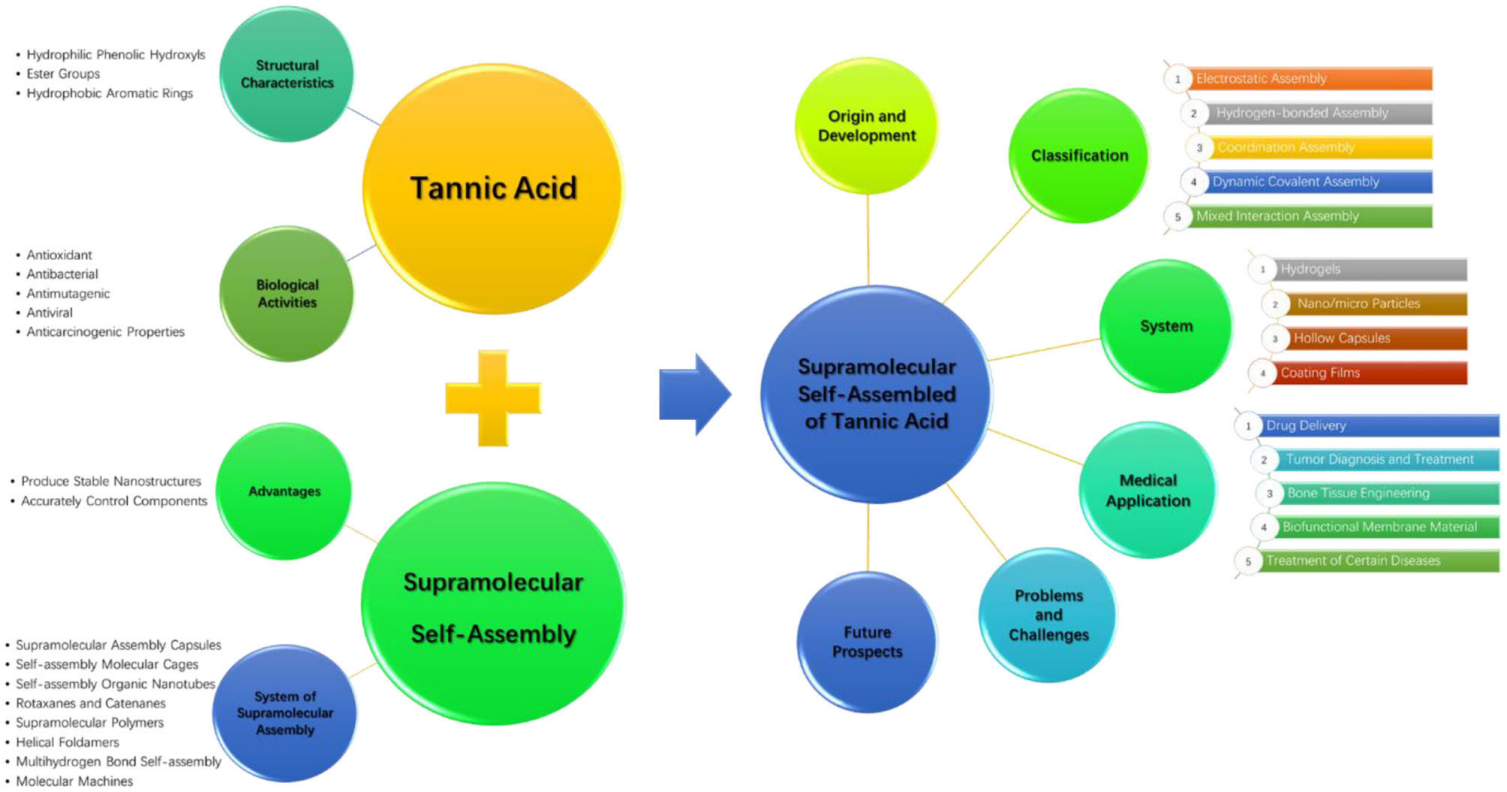
The comprehensive summary of the review.

In this review, combined with the structural characteristics and biological activity, we introduce the principle and development process of TA supramolecular assembly. The latest progress in TA supramolecular assembly and its biomedical applications are reviewed in two different dimensions including the supramolecular assembly driving force and the assembly system. In addition, we discuss the challenges associated with TA supramolecular assembly and its developmental prospects. The comprehensive summary of the review is graphically shown in Figure 1.

## Overview of Supramolecular Assembly

With the in-depth understanding of molecular chemistry based on chemical bonds, it has been found that traditional molecules cannot perform certain specific, advanced, or complex functions but need an organized multimolecular system to work cooperatively. Therefore, it is necessary to research the interaction between molecules at the molecular level, so the concept of supramolecular chemistry has been proposed. Supramolecular chemistry works with the interaction between molecules, including ion–ion interactions, ion–dipole interactions, dipole–dipole interactions, van der Waals forces, tight packing in solids, hydrophobic effects, hydrogen bonding, π-π stacking of aromatic rings, cation–π interactions, and anion–π interactions. The study of supramolecular chemistry originated from Pedersen's report about the synthesis of crown ethers and their complexation properties of alkali metal ions in 1967 (Pedersen, 1967, 1988). Additionally, in 1978, Lehn clearly put forward the concept of supramolecular chemistry, pointing out that supramolecular chemistry concerns molecular assemblies and intermolecular bonds (Lehn, 1978). In fact, the cleavage and formation of both covalent bonds in molecular chemistry and non-covalent bonds in supramolecular chemistry are the flow and redistribution of electron clouds, so they are essentially the same. Molecular recognition, an important means of supramolecular chemistry, is a process by which a specific host molecule selectively binds to a guest to produce specific functions (Lehn, 1973), so its binding is purposeful. Self-assembly is an important method for the synthesis of supramolecular interactions. Based on specific molecular recognition modes, self-assembly is a process by which molecules or other assembly elements spontaneously form ordered structures via weak interactions (Philp and Stoddart, 1996; Lehn, 2002; Whitesides and Grzybowski, 2002). The emergence and application of self-assembly have greatly improved the efficiency of constructing complex host molecules. Relatively simple monomers can be synthesized by self-assembly, and the binding sites can be encoded in the monomer structure. Monomers rely on the recognition of bonding sites to achieve “procedural” self-assembly (Lehn, 2000), spontaneously forming complex, highly selective, reversible host molecules. In addition, the host molecule for self-assembly can change its structure according to the external stimulation or guest to achieve the maximum degree of bonding. In the field of supramolecular chemistry, we call it supramolecular self-assembly. Supramolecular self-assembly is a process of spontaneous molecule formation of unique nanostructures via non-covalent intermolecular interactions (Wang et al., 2018b) and dynamic covalent interactions (Caulder and Raymond, 1999).

**Figure 2:**
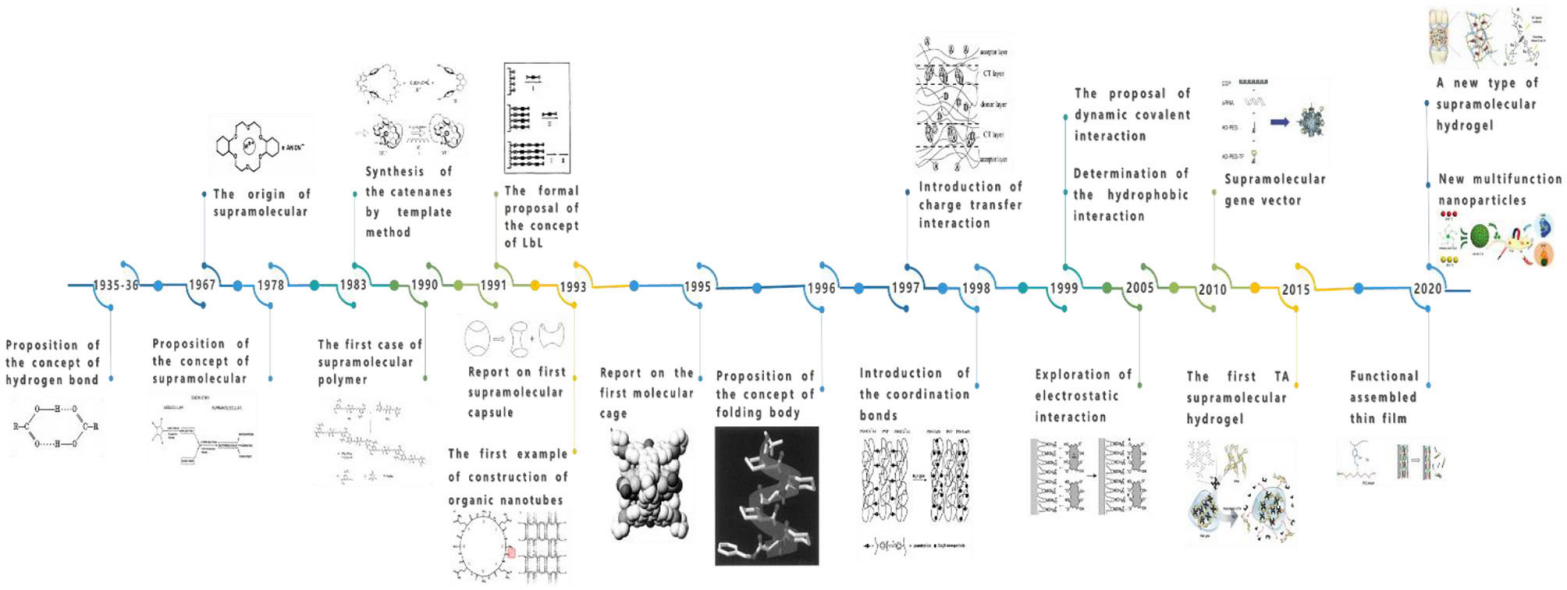
The origin and development of supramolecular assembly.

With the deepening of research, supramolecular assembly has intersected with other disciplines, and some new research fields continue to emerge. According to basic research, a near-perfect system of supramolecular assembly has emerged. Additionally, supramolecular assembly has made some progress in the fields of supramolecular assembly capsules, self-assembly molecular cages, self-assembly organic nanotubes, rotaxanes and catenanes, supramolecular polymers, helical foldamers, multihydrogen bond self-assembly, and molecular machines, among others. The origin and development of supramolecular assembly are shown in Figure 2.

Early studies of molecular containers have focused on carcerands and hemicarcerands, but the synthesis of these molecules is complex, and releasing guest molecules is not easy. Therefore, scientists have gradually turned their attention to reversible supramolecular capsules, which are self-assembled by relatively simple monomer molecules via weak non-covalent interactions between molecules. The supramolecular assembly capsule originated from the first hydrogen-bonded supramolecular capsules with a tennis ball shape based on the glycoluril structure, as reported by Wyler et al. (1993). This field is still in the stage of vigorous development. However, the design and construction of supramolecular capsules require the consideration of various factors, and the selection of guests requires comprehensive consideration of various factors such as shape, size, and surface chemical properties. Thus, the kinds of capsules available are few thus far, and basic research persists; therefore, a long time is needed for its industrialization and commercial application.

The molecular cage is the assembly of molecules with a three-dimensional cage cavity structure, which usually has a complex structure. In the 1990s, Fujita reported a new nanoscale metal-bonded molecular cage based on PdL2-based building blocks (Fujita et al., 1995). With the development of supramolecular assembly, molecular cages have attracted interest and attention because of their unique structural characteristics and wide range of potential applications. An increasing number of metal-bonded self-assembled molecular cages, covalently bonded molecular cages, and other self-assembled molecular cages have been synthesized, which are widely used in the fields of molecular recognition, catalysis, and gas adsorption, among others.

As a kind of one-dimensional nanomaterial, carbon nanotubes have unique physical and chemical properties. Since carbon nanotubes were discovered by Iijima (1991), after nearly 30 years of development, they have been chemically modified to different degrees and endowed with different properties, although some limitations persist in the modification of carbon nanotubes with only one element, such as difficulty in controlling the size, derivation after synthesis, and responses to external stimuli. Supramolecular self-assembled organic nanotubes can overcome these shortcomings and thus facilitate the functionalization, derivatization, and reversible response to external environmental changes, leading to the wide use of supramolecular self-assembled nanotubes in the fields of molecular inclusion and separation, chemotherapy, transmembrane transport, ion channels, and drug delivery, among others. The Granja team successfully constructed the first case of self-assembled organic nanotubes using cyclic peptides, synthesizing cyclic octapeptides with an alternating arrangement of α-(d,l)-amino acid residues and thus opening a new world for the development of self-assembled organic nanotubes (Ghadiri et al., 1993).

Both rotaxanes and catenanes are very interesting compounds that link the subunits of the molecular structure by the mechanical force of non-covalent bonds. A supramolecular assembly usually appears in the construction process of rotaxanes and catenanes, the structure of which is similar to rotaxanes, but the linear molecules have no capped groups, or the capped groups are too small to block macrocyclic molecules, which are called quasi-rotaxanes. The special structures of quasi-rotaxanes, rotaxanes, and hydrocarbons endow them with great application potential in the construction of molecular devices and nanofunctional materials. The interlocking structures of rotaxanes and catenanes were initially synthesized via a statistical method. There is no mutual attraction between the subunits of the interlocking molecules, and the interlocking structure is formed because of the probability that the two randomly pierced parts cannot be separated after the reaction. In 1960, Wasserman first obtained the interlocking ring, called the catenane, with a yield of < 1% by statistical methods (Wasserman, 1960). In 1967, I. T. Harrison and S. Harrison first described the rotaxanes, which they called hooplanes (Harrison and Harrison, 1967), and the yield was only 6%. Obviously, the probability of random piecing together of subunits is small, so the yield of interlocking molecules is low. In 1983, (Dietrich-Buchecker et al., 1983) formed a stable quasi-rotaxane complex by ion coordination and then synthesized hydrocarbons with a yield of 27% by the cyclization reaction. Sauvage pioneered the method of synthesizing catenanes using univalent copper ions as templates and efficiently synthesized interlocking molecules by the template method. In this method, copper ions coordinate with organic ligands to gather two molecules together, and then the ring-closing reaction is used to connect the terminal to form the ring–ring catenane structure (Dietrich-Buchecker et al., 1983). Thus, the yield can be greatly improved by using the non-covalent weak interaction between molecules as a template to prepare interlocking structures.

The supramolecular polymer is the molecular aggregate assembled by non-covalent bond interactions between monomer structural elements (De Greef et al., 2009). They are mostly monomers with bifunctional groups formed spontaneously by molecular self-assembly in suitable solvents without any initiator. In 1990, Lehn, a famous supramolecular chemist, reported the first case of the supramolecular polymer (1.2)_n_ based on hydrogen bonding, which is the first case of supramolecular polymer (Claudine Fouquey and Anne-Marie, 1990). However, the non-covalent interaction in the supramolecular polymer structure lacks directionality, which can easily lead to a microphase separation structure or gelation in the process of polymer network formation. Therefore, how to incorporate sufficiently strong but still reversible interactions has become a difficult problem for researchers. Sijbesma et al. (1997) synthesized reversible self-assembling polymer systems, using units of 2-ureido-4-pyrimidone that dimerize strongly in a self-complementary array of four cooperative hydrogen bonds as an associating end group to solve the problem of polymer reversibility and high strength. They also pointed out that thermal and environmental controls on life and bonding strength can adjust the properties of polymers so that polymer networks with thermodynamically controlled structures can be formed, which has great significance in the development of reversible supramolecular polymers (Sijbesma et al., 1997). Because of the reversibility of non-covalent bonds, the polymerization and depolymerization of supramolecular polymers can easily occur; thus, as an excellent intelligent material, it has wide application prospects in sustained drug release. For example, Davis' team reported the first clinical trial using supramolecular cyclodextrin as a gene carrier, which uses cyclodextrin polymer and siRNA to form gene nanoparticles to treat patients with pigment cancer by targeting, and the effect of clinical gene therapy was achieved (Davis et al., 2010). Although supramolecular polymers have been studied for decades, they are still in the early stage of development, including the principle of monomer self-assembly, the mechanism of supramolecular polymerization, and the relationship between structure and function.

Protein is the most abundant and functional polymer in cell components, which plays the role of an executor of various life functions in life activities. To perform their biological function, proteins must be correctly folded into a specific configuration. These orderly and stable folding structures are mainly realized via the cooperation of non-covalent bond interactions. In recent years, because of the great interest in simulating the helical structure of biopolymers, the study of folded molecules has become a research hotspot in the field of supramolecular self-assembly. On the one hand, folded molecules play a vital role in simulating the structure of biopolymers, which is expected to provide theoretical and scientific bases for the synthesis of proteins; on the other hand, folded molecules can form a spiral structure with nanocavities or a new type of organic nanotubes by self-assembly. They have potential applications in molecular recognition, ion channels, molecular catalysis, and nanoreactors, among others. In recent years, various types of oligomers have been synthesized. These oligomers adopt helical secondary structures via intramolecular non-covalent interactions, and they are called foldamers together with other solid polymers with secondary structures. In 1996, (Appella et al., 1996). first proposed the term “foldamer” (Appella et al., 1996), but research on foldamers has existed for a long time. In 1998, Gellman reported the original definition of the foldamer: any polymer with a specific compact structure (Gellman, 1998). Because of the inaccurate use of the word “polymer” in this concept, what are widely studied are not polymers but oligomers with a specific molecular formula and relative molecular mass, and the term “specific compact structure” in the concept is too vague. Therefore, in 2001, (Hill et al., 2001) made necessary modifications to the definition of foldamer and defined it as an oligomer that presents an ordered and compact conformation in solution; the tight conformation is stabilized by direct non-covalent interactions of non-adjacent units (Hill et al., 2001). This definition is more accurate than the previous one, showing that the oligomer changes from disorder to order in solution; that is to say, the driving force of the “folding reaction” comes from the potential non-covalent bond interaction of the oligomer. Therefore, the oligomer that cannot undergo a “folding reaction” cannot be called a foldamer. In recent years, biomimetic folding based on small molecules has attracted the attention of researchers. For example, Xiao et al. (2019a) dimerized small organic molecules through quadruple hydrogen bonds and then formed folding dimers with the help of interaction, and they can further form one-dimensional supramolecular stacking through additional weak interactions in the solid state, which is similar to the biomimetic folding of DNA. Hydrogen bonding is a very important non-covalent interaction in supramolecular chemistry, and it is called the “omnipotent interaction in supramolecular chemistry” in the variety of non-covalent interactions. Because of the characteristics of high strength and remarkable orientation, hydrogen bonding has been widely used in the field of supramolecular assembly in recent years. In 1920, Latimer and Rodebush (1920) first described the phenomenon of the hydrogen bond clearly when they explained the association between water molecules. Bernal and Megaw (1935) and Huggins (1936) formally proposed the concept of the hydrogen bond in 1935 and 1936, respectively. Hydrogen bonding means that when H forms a covalent bond with F, O, N, and other atoms, these atoms are partially negatively charged because of their high electronegativity and attraction to electrons, while the hydrogen atom is partially positively charged, forming a state similar to that of hydrogen ions and thus attracting lone pairs of electrons of F, O, N, and other atoms adjacent to it. This concept is widely accepted because of Pauling's (1985) famous monograph on chemical bonds. Although the energy of a single hydrogen bond is weaker than that of a covalent bond, the synergistic effect of multiple hydrogen bonds can reach a comparable level. Designing and synthesizing a simple, highly stable, and easy-to-derive multihydrogen bond system will be the focuses of research in the future.

The molecular machine, an independent molecule or molecular assembly, extends the concept of a macroscopic machine to the molecular level and can perform a machine-like motion under appropriate external stimulation (Balzani et al., 2000). One of the important goals of supramolecular assembly is to create orderly, functional devices at the molecular level that can interfere with, store, process, and transmit information. There are many such exquisite machines in nature; for example, both the photocapture antennae of bacteria formed by photosynthesis and ATP synthetase are formed by a number of molecules via self-assembly and self-organization. Supramolecular self-assembly is an effective tool to construct molecular machines. A molecular machine based on a single molecule involves changes in the molecular conformation or configuration around the covalent bond when its components move. For example, (Pollard et al., 2007) designed molecular motors via Zmure isomerization with a double-bond configuration (Pollard et al., 2007). There are essential differences between molecular machines based on supramolecular assembly and a single molecule. The motion in molecular machines based on supramolecules often involves the change in the conformation of supramolecular systems. Through the method of self-assembly, selecting the appropriate construction unit, and controlling the manner of non-covalent interactions, the ideal molecular assembly can be obtained under the drive of thermodynamics. However, regardless of structure or function, the complexity and ingenuity of biomolecule machines are far beyond synthetic molecular machines. The study of synthetic molecules is still in its infancy, and some problems must be further explored. For example, most studies on the behavior of molecular machines are carried out in solution, and it is necessary to arrange them in a certain, orderly way to function at the macrolevel similar to dynamic proteins, including tissue at the interface, deposited on the surface, and fixed on the membrane or porous materials.

The bottom-up construction of nanoscale functional materials by supramolecular self-assembly is a hotspot and challenge in current research, and it is an important means to create new materials and produce new functions. Compared with the traditional chemical reaction, the supramolecular assembly system has some typical characteristics. The first is order, wherein the structure of self-assembly is more orderly than individual components. Second, in terms of interaction forces, non-covalent bonds and dynamic covalent bonds are used as driving forces in supramolecular assembly, and these weak interactions are far smaller than traditional chemical bonds, but they determine the physical properties of liquids, the solubility of solids, and the molecular assembly of biofilms, so they play an important role in material synthesis. Finally, in terms of the construction unit, the construction unit of supramolecular assembly includes not only atoms and molecules but also nanoscale and micron structures with different chemical compositions, shapes, and functions. Supramolecular assembly usually involves a layered assembly process, which is called layer-by-layer (LbL) assembly. LbL is one of the classic supramolecular assembly techniques, with its main advantages including simplicity, low cost, ruggedness, and flexibility (Decher et al., 1992). Additionally, multifunctional supramolecular nanomaterials are obtained by accurately regulating the assembly process at the nanometer scale (Yang et al., 2019b). In the past half-century, LbL assembly has received increasing attention with the promotion of existing materials and assembly technology and characterization of technological innovation.

## Structural Features of TA Contributing to Self-Assembly

**Figure 3:**
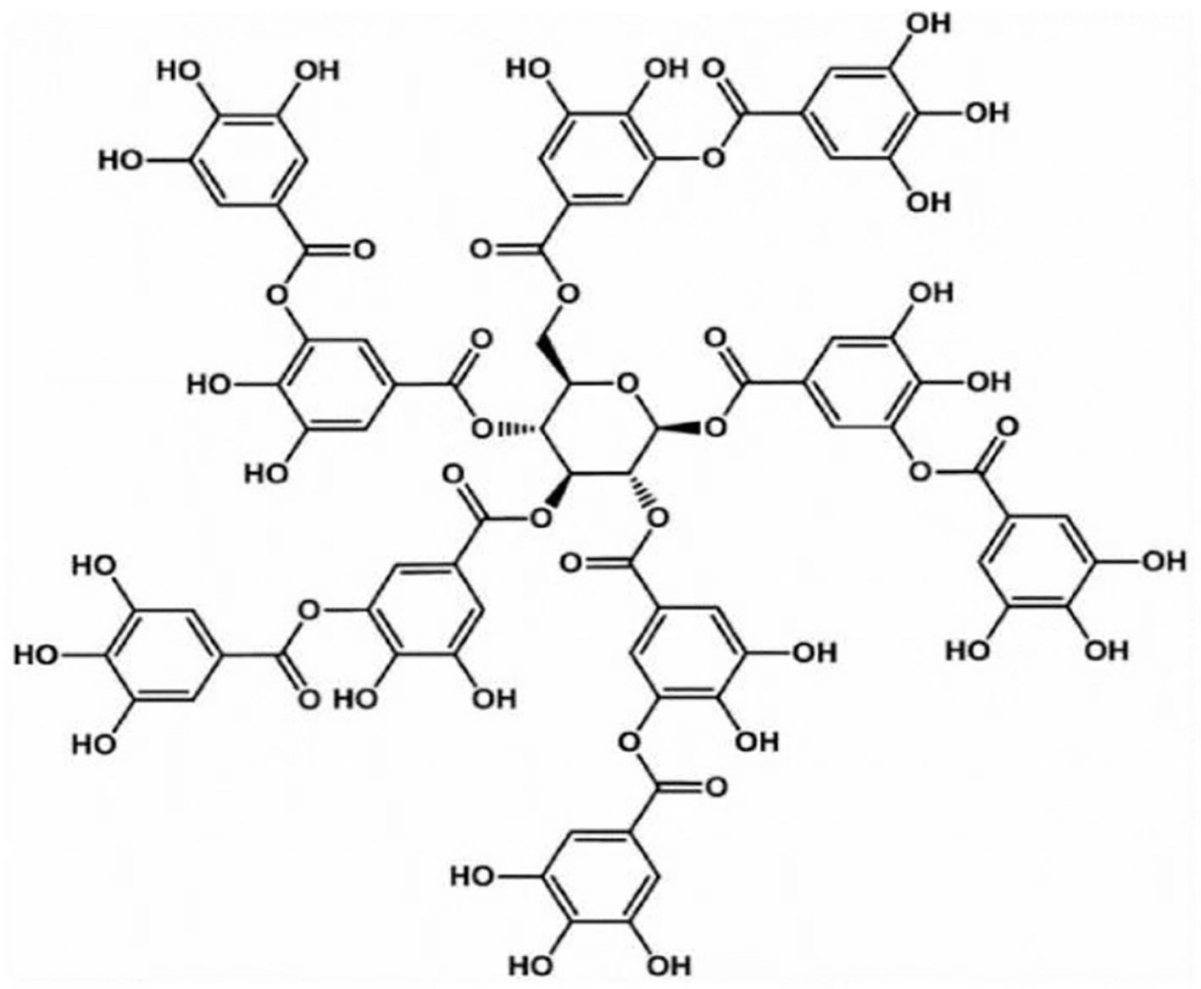
The molecular structure of TA.

TA, as shown in Figure 3, which is mostly distributed in various plants containing vegetables, fruits, oak, grapes, tea leaves, olives, and others, is a biodegradable, naturally existing polyphenolic compound (Wu et al., 2015b; Shin et al., 2018), and it has been affirmed as a safe potential direct food-product additive by the US Food and Drug Administration (Le et al., 2018). TA, as a well-known high-molecular-weight polyphenolic compound and hydrolyzable tannin (Shutava et al., 2005), has antidiarrheal, convergence, and hemostatic functions (You et al., 2014). As a classical polyphenol, TA often exists in solution as loosely bounded complexes due to its ability to form intramolecular and intermolecular hydrogen bonds (Shutava et al., 2005). Moreover, TA has also been revealed to exhibit high biological activities such as antioxidant, antibacterial, antimutagenic, antiviral, and anticarcinogenic properties (Costa et al., 2011; Le et al., 2018). These characteristics of high bioactivity, extensive sources, low expense, and good biocompatibility make TA widely used in the leather manufacturing, food processing, and medical and biological applications industries (Wu et al., 2015b; Abouelmagd et al., 2019). In particular, its application in biology and medicine has aroused the interest of scientists.

The peculiar properties of TA mainly depend on its unique structural characteristics. According to structural analysis, TA possesses a central glucose molecule, which is linked to 10 gallic acid units via five diacetyl ester groups (Abouelmagd et al., 2019). Abundant adjacent phenolic hydroxyls and ester groups provide TA with high chemical reactivity, providing sufficient response sites to different substances (Gao and Zharov, 2014; Xu et al., 2019), which facilitate the ideal properties of TA as a sustainable scaffold on substrate surfaces. In addition, the unique structural properties of TA, including abundant hydrophobic aromatic rings and hydrophilic phenolic hydroxyl groups, allow it to react with various substances through covalent and non-covalent interactions, such as hydrogen bonding and electrostatic, π-π stacking, and hydrophobic interactions (Song et al., 2019; Zhao et al., 2019b). For example, the interaction between TA and ammonium cations can be attributed to electrostatic interactions, hydrogen bonding, and π-π stacking (Gao and Zharov, 2014). TA also has excellent antioxidant properties due to a large number of phenol groups because it can scavenge reactive oxygen species (ROS) by converting the phenol to the quinone group in an oxidative stress environment (Chung et al., 1998b). Moreover, the weak acidity and extensive hydrogen bonds of TA are reasonable because it contains 25 hydroxyl groups (Richardson et al., 2016; Onat et al., 2018). However, the pKa of TA varies for different sources, with an average close to 8.5, and is easily affected by oxidation in basic solutions (Ringwald and Ball, 2016). In addition, TA has not only an effective adsorption performance but also immunomodulatory effects in animals (Takemoto et al., 2015).

In recent years, surface modifications and structural functional materials based on TA have become a research focus. For example, TA has been used to obtain coating by reaction with Fe^3+^ ions on various substrates within only 30 s, where a modified TA/Fe^3+^ coating sealed the microholes of a filter membrane with a geometry similar to dentinal tubules (Oh et al., 2015). TA is also conducive to enhance the antibacterial and antioxidant activities of sponges (Shukla et al., 2012). By adding weak polyelectrolytes in the film assembly, the obtained PEM film is able to preprogram adjustable retention/release properties (Zhuk et al., 2014), which can be used to regulate drug release. Previous work has demonstrated that hydrophobic interactions of TA and proteins contribute to enhancing their mechanical and thermal properties. Related reports have revealed that TA can precipitate not only various proteins, including collagen, gelatin, and albumin, but also some polysaccharides and alkaloids (Aoki et al., 1981; Shutava et al., 2005). It also interacts with certain specific and targeted amino acids (Fraga et al., 2010). The LbL films fabricated from TA and PVPON via reversible hydrogen bonding are used to controllably regulate physiological processes by gradually disassembling and releasing TA into the media (Zhou et al., 2013). The films compounded by TA and lignin are expected to enhance Col-H strength against compression force, the antibacterial effect, and mechanical stability (Velmurugan et al., 2014; Gogoi et al., 2017). TA is increasingly used in biomaterials and medicine because of its excellent biocompatibility and high reactivity.

## Development of TA Self-Assembly

### Self-Assembly Principle of TA

**Figure 4:**
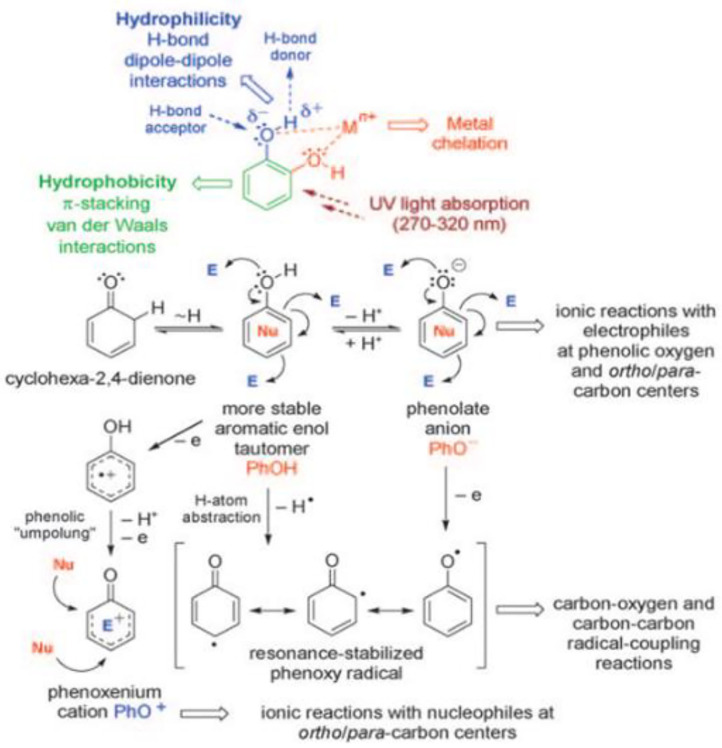
Basic reactivity of the phenol [E = electrophile, Nu = nucleophile (Quideau et al., 2011)].

Supramolecular assembly of TA depends on the physical and chemical properties of phenol functional groups in TA, which endows TA with a variety of reaction activities, as shown in Figure 4. Phenol functional groups in TA molecules can be used as hydrogen donors or hydrogen receptors to participate in various chemical reactions (Quideau et al., 2011). Because phenol is a kind of molecule with stable enol tautomerism, the weak nucleophilic property of phenol changes to a strong nucleophile when the hydroxyl on phenol is deprotonated to phenol anion (PhO^−^). As a result, TA can be used as an oxygen or a carbon-based nucleophile to participate in multiple ion reactions (Quideau et al., 2011). Phenol cations (PhO^+^) can be formed by two-electron transfer dehydrogenation coupling oxidation under neutral or weakly acidic conditions, and this delocalized and stable cationic intermediate is a strong carbon-based electrophilic, so it is easy to react with nucleophiles. Moreover, the amphiphilic nature of this complex structure of the TA—hydrophobic aromatic ring and hydrophilic phenolic hydroxyl group—endows TA with supramolecular assembly ability through hydrophobic interaction. The planar aromatic nuclei of phenol give TA the ability to assemble supramolecularly through π-π stacking (van der Waals force). Because polyphenol hydroxyl groups are negatively charged under physiological pH, TA is expected to achieve supramolecular assembly by electrostatic interaction with cationic compounds (Abouelmagd et al., 2019). Moreover, the oxygen atom with strong electronegativity can be used as a hydrogen acceptor, while the hydrogen atom with weak electronegativity serves as a hydrogen donor in the polar O–H bond of the polyphenol hydroxyl group. Thus, TA has the ability to undergo supramolecular assembly through hydrogen-bond interactions. In addition, the two adjacent phenolic hydroxyl groups of TA are able to coordinate with metal ions to form complex precipitates via the formation of oxygen anions. TA is able to supramolecularly assemble through metal coordination interactions due to the abundant catechol and pyrogallol in its structure (Andjelkovic et al., 2006).

### The Origin and Development of LbL Assembly of TA

The LbL assembly method plays an important role in the supramolecular assembly of TA, which is the alternating deposition of interacting materials on different substrates. The strategy of alternating deposition can be traced back to the pioneering work carried out by Iler and Kirkland in 1964–1966 (Kirkland, 1965; Iler, 1966), who prepared inorganic films by the assembly of negatively charged silica particles and positively charged alumina fibers. However, it was not until 1991 that Decher and Hong, as pioneer researchers, carried out a comprehensive characterization of LbL multilayer films (Decher and Hong, 1991), which successfully opened the door for LbL assembly. In the traditional sense, LbL assembly involves alternating deposition through electrostatic interactions driven by enthalpy and entropy (Fu and Schlenoff, 2016). However, after Cassier et al. (1998) finished their preliminary study of biotin–streptavidin interactions, the driving forces of LbL assembly were expanded to other interactions rather than simplex electrostatic interactions. For example, Stockton and Rubner (1997) first conducted a groundbreaking study on the preparation of LbL modules using hydrogen-bond interactions; coordination bonds (Xiong et al., 1998) and charge transfer interactions (Shimazaki et al., 1997) were introduced in 1997 and 1998, respectively. Kotov (1999) and Cochin and Laschewsky (1999) identified hydrophobic interactions as the main driving forces for the buildup of LbL in 1999. In addition, host–guest interactions are also widely used in LbL assembly of multilayer films in biomimetic systems (Muller et al., 1993). In brief, supramolecular nanomaterials with different properties and functions can be obtained by different assembly interactions. In addition, the supramolecular assembly based on TA can be traced back to the electrostatic interactions involved in the zeta potential and surface charge density of nylon 6 fibers treated with acid dye solution by Ogasawara et al. (1981). It was not until 2005 that Shutava et al. (2005), formally explored the electrostatic assembly of TA and cationic polymers, which opened a new world for LbL assembly of supramolecular materials based on TA.

### Classification of TA Self-Assembly

According to the assembly driven interaction, TA self-assembly can be divided into four major types: (1) electrostatic assembly, (2) hydrogen-bonded assembly, (3) coordination assembly, (4) dynamic covalent assembly, and (5) mixed interaction assembly.

### Electrostatic Assembly of TA

**Figure 5:**
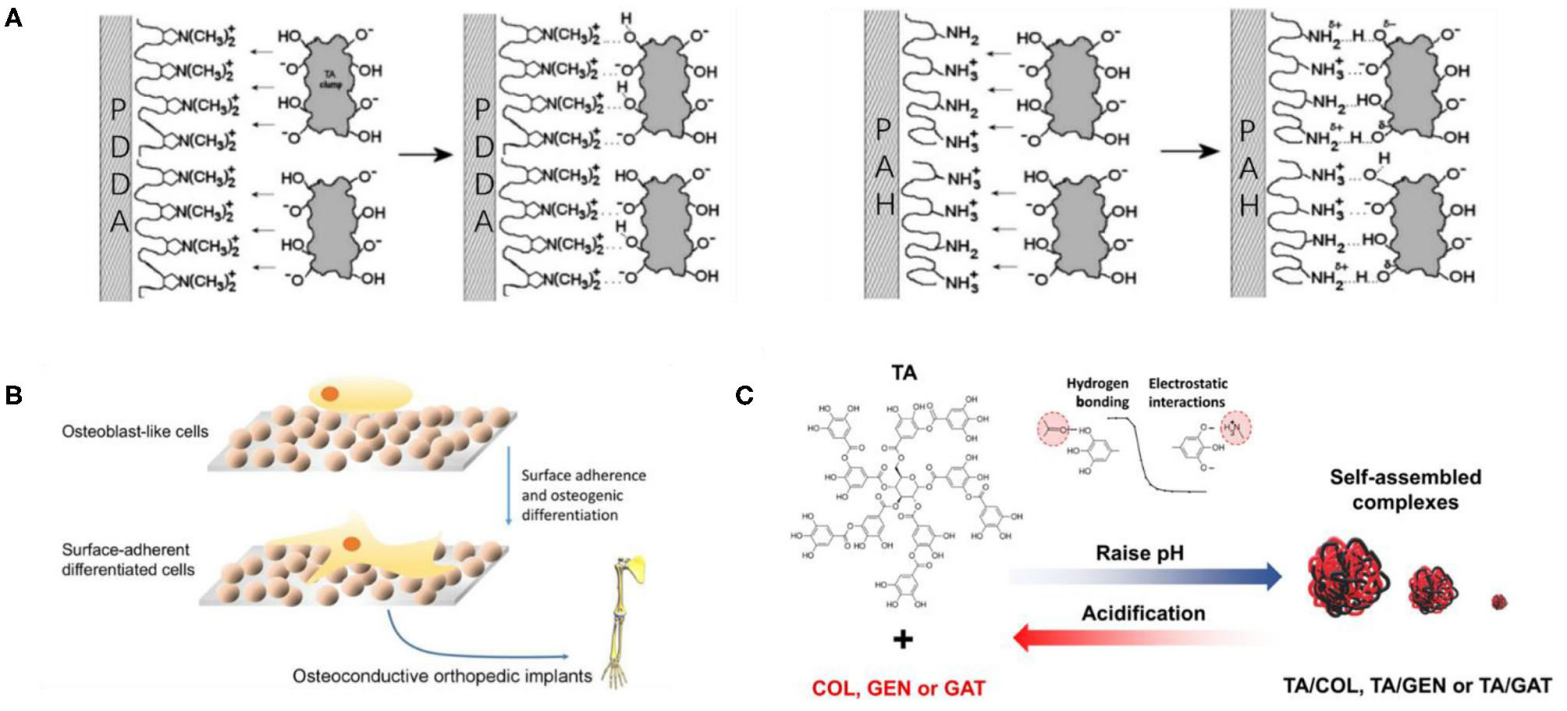
Electrostatic assembly and application of TA. **(A)** Possible bond formation models of the interaction between TA and polyamine in LbL films for PAH and PDDA (Shutava et al., 2005). **(B)** Osteoconductive behavior of PHPE-TA multilayers (Onat et al., 2018). **(C)** The pH-dependent assembly and dissociation of TA/drug complexes driven by electrostatic interactions and hydrogen bonding (Abouelmagd et al., 2019).

The electrostatic assembly of TA is the supramolecular assembly process by electrostatic interaction between TA as a negatively charged assembly unit and positively charged polymer. The electrostatic assembly of TA is based on the ionization of abundant polyphenol hydroxyl groups in the TA molecule, filling its surface with a negative charge. Positively charged polymers are generally cationic polymers, which have a strong interaction with negatively charged cell membranes under physiological pH. They can adhere to the cell surface and enhance penetration, so they are attractive in the field of biomedicine. Shutava et al. (2005) performed a groundbreaking study on the electrostatic assembly of TA and cationic polymers. They alternately assembled TA with two different cationic polymers including strong poly(dimethyldiallylamide) (PDDA) and weak poly(allylamine) (PAH), as shown in Figure 5A, to obtain nanomembranes and capsules with a pH response. The capsules showed low permeability at pH 5–7 and high permeability at low or high pH. Combined with the antioxidant, antibacterial, and antiviral properties of TA, the pH-responsive capsules had the potential to be used as drug carriers. However, Shutava et al. (2005) did not carry out cytotoxicity experiments on the obtained materials, and the high density of positive charge on most cationic polymer functional groups may cause cytotoxicity, so it remains doubtful whether the materials can be used in drug delivery. Although non-cytotoxic cationic polymers are limited, poly(4-hydroxy-l-proline ester) (PHPE) is a non-cytotoxic cationic polymer, which is cationic under physiological pH. Its natural degradation product is hydroxyproline (Hyp), which can be safely considered for biomedical applications. Onat et al. (2018) prepared water-soluble complexes of PHPE and TA (PHPE-TA) at pH 4 and deposited the film on the surface by LbL assembly, followed by crosslinking the multilayer film with NaIO_4_ to enhance stability under physiological conditions. Because the prepared LbL film shows good bone conduction properties, it is expected to be used for the coating of orthopedic implants, various biomaterials (such as bone screws and knobs), or special scaffolds for bone tissue engineering, as shown in Figure 5B. In addition, based on TA maintenance of antibiotic molecules through electrostatic interactions, the film has biological response characteristics, which can broaden the spectrum of antibacterial activity. Zhuk et al. (2014) used LbL assembly to directly assemble TA with several cationic antibiotics (tobramycin, gentamicin, and polymyxin B) to form a highly efficient, bioresponsive, controlled-release antibacterial coating, which can be used to prevent bacterial colonization in biomedical devices. In fact, because of the complexity of the antibiotic structure, there is hydrogen-bond interaction between TA and antibiotics in addition to electrostatic interactions. Recently, Abouelmagd et al. (2019) used the complex of TA and cationic antibiotics as a new pH-responsive drug carrier with high drug loading and optimal stability, indicating that TA/antibiotic self-assembly complex is a good carrier for pH-sensitive water-soluble drugs, as shown in Figure 5C. Although the TA self-assembly materials based on electrostatic interaction show certain advantages in the biomedical field, there are still some problems that limit the further development of TA supramolecular assembly. First, electrostatic-based assembly is sensitive to the ionization state of salts and polymers, and they are relatively unstable without covalent crosslinking (Hoogeveen et al., 1996). Second, selectable and preparable materials for electrostatic interaction are limited, which are suitable only for the preparation of charged and water-soluble multilayer materials that are sensitive to external stimuli (Xiao et al., 2016). Finally, the introduction of cationic polymers in electrostatic interaction may cause cytotoxicity.

### Hydrogen-Bonded Assembly of TA

**Figure 6:**
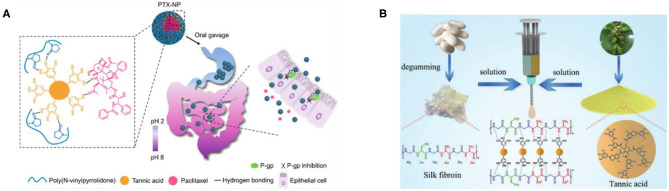
Hydrogen-bonded assembly and application of TA. **(A)** PTX-NP assembled by intermolecular hydrogen-bonded interactions have features including P-gp inhibitory functions and pH-sensitive behavior (Le et al., 2018). **(B)** The formation schematic of the FT hydrogel adhesive (dominated by hydrogen-bond interactions) (Luo et al., 2020).

Compared with electrostatic assembly, hydrogen-bonded assembly is more attractive for biomedical applications because toxic cationic polymers are not introduced in the assembly process (Takemoto et al., 2015). However, the stability of hydrogen-bonded assembly under physiological conditions limits its practical application. The natural polyphenol TA with a multibranched chain can provide relatively stable hydrogen bonding under physiological conditions (Sundaramurthy et al., 2014), so the hydrogen-bond assembly of TA has been widely studied. Le et al. (2018) used paclitaxel (PTX) as a model drug and prepared PTX-loaded TA/poly (N-vinylpyrrolidone) nanoparticles (PTX-NP) by flash nanoprecipitation via the intermolecular hydrogen-bond interactions, and they explained the assembly mechanism of hydrogen-bonded PTX-NP by molecular dynamics simulation. Moreover, it has been confirmed that PTX-NP may be a promising oral drug formulation for chemotherapy *in vitro* and *in vivo*, as shown in Figure 6A (Le et al., 2018). Adatoz et al. (2018) prepared hydrogen-bonded and pH-responsive poly(2-ethyl-2-oxazoline) (PEOX) and TA multilayers by LbL deposition, which can be reassembled into H-bonded pH-responsive PEOX/TA fibers in phosphate buffer solution, pH 3, validating that pH-responsive fiber aggregates have certain application prospects in a variety of biomedical applications from controlled release to sensors. Luo et al. (2020), using silk fibroin (SF) and TA as raw materials, effectively constructed multifunctional hydrogel adhesives with high extensibility (up to 32,000%), real-time self-healing ability, underwater adhesion, sealing, biocompatibility, and antibacterial properties, through the hydrogen bonding of TA and SF chains in water, which has potential applications in medical fields such as tissue adhesives and integrated bioelectronics, as shown in Figure 6B. Although the polyhydroxyl structure of TA can provide relatively stable hydrogen bonds under physiological conditions due to the inherent limitations of the hydrogen-bond structure, it is greatly affected by pH, ionic strength, and other factors. As a result, the hydrogen-bond assembly of TA is unstable compared with the electrostatic interactions.

### Coordination Assembly of TA

**Figure 7:**
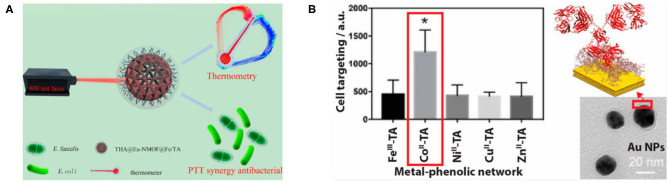
Coordination assembly and application of TA. **(A)** The application of THA@Eu-NMOF@Fe/TA nanoprobe assembled by coordination (Yan et al., 2019). **(B)** The MPN coatings assembled by crosslinking of metal ions (FeIII, CoII, NiII, CuII, or ZnII) with TA can be used to enhance targeting (Zhang et al., 2020). *means the highest median Au intensity.

The coordination assembly of TA occurs through the MPN structure to form metal–organic supramolecular assembly materials (Richardson et al., 2016). At present, more research is focused on the assembly of TA with iron or ferrous ions, which has many applications in the field of biomedicine such as antibacterial coating (Ko and Huang, 2020), clinical diagnosis, nanoprobe treatment (Zou et al., 2018; Yan et al., 2019), tooth desensitizers (Zhou et al., 2013), separation and protection of living cells (Kim et al., 2017), self-healing and functional films (Lee et al., 2018), and antioxidant coatings (Maerten et al., 2017). Ejima et al. (2013) first proposed the synthesis of coordination complexes using natural polyphenol TA as an organic ligand and Fe(III) as an inorganic crosslinking agent, which can be used to prepare various films and particles on a series of substrates by one-step assembly. Because TA can be used as a molecular tool for chelating and developing iron activation, TA as a natural iron complexing agent may be a promising method for the prevention and treatment of iron-related cancer or other iron overload diseases. For example, Phiwchai et al. (2018) found that the self-assembled Fe^3+^/TA complex formed by TA binding with extracellular iron has the characteristics of autophagy induction. Yan et al. (2019) designed and assembled an intelligent integrated nanoprobe (THA@Eu-NMOF@Fe/TA) based on the Eu(III) nanometal–organic skeleton (Eu-NMOF), in which photothermal therapy (PTT) and antimicrobial agents are carried out simultaneously by using coordinated complex Fe/TA as a photothermal and antibacterial agent. This opens the way for a new type of cancer treatment probe to achieve real-time temperature sensing feedback in the process of PTT and antibacterial effects, as shown in Figure 7A (Yan et al., 2019). In addition, TA can be complexed with other metals such as gold, silver (Fang et al., 2018b), zirconium (Bag et al., 2015), and stainless steel (Xu et al., 2019) to obtain supramolecular nanomaterials with different properties and biomedical functions. Zhang et al. (2020) cross-linked TA with metal ions such as [Fe(III), Co(II), Cu(II), Ni(II) or Zn(II)] to form an MPN coating, followed by coating with gold nanoparticles, and physically adsorbed antibodies on the surface, demonstrating that it could enhance the targeting of antibodies and their respective antigens, as shown in Figure 7B. Although the coordination complex of TA can prepare many biological functional materials, it still has some limitations. For example, the materials coordinated with TA need specific metal elements. Therefore, coordination assembly is limited by material selection (Zou et al., 2018). In addition, the assembly process is slow because of the influence of metal ligands and solvent types, and the supramolecular nanomaterials constructed may have some impurities.

### Dynamic Covalent Assembly

**Figure 8:**
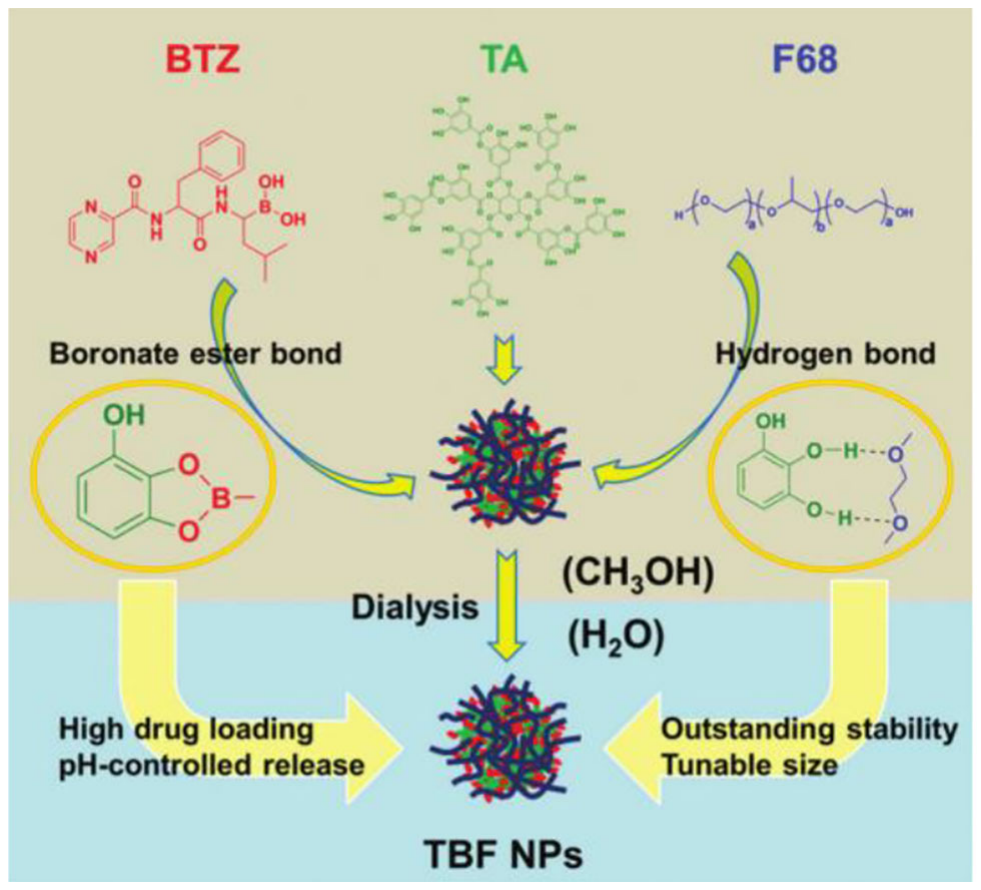
Dynamic covalent assembly and application of TA. Construction and properties of a dynamic self-delivery system based on tannic acid (TA), bortezomib (BTZ), and poloxamer (F68) (Zhao et al., 2019a).

The dynamic covalent bond integrates the characteristics of the covalent bond and non-covalent bond, endowing it with special features (Cougnon and Sanders, 2012; Lehn, 2012; Li et al., 2013). They can be formed and broken reversibly, similar to non-covalent bonds, whereas they can be as strong and durable as covalent bonds under different conditions. In recent years, because dynamic covalent interactions combine the robustness of “classical” covalent bonds with the typical flexibility of non-covalent interactions accelerating the development of complex nanostructures, increasing attention has been focused on supramolecular chemistry (Wilson et al., 2014). Five widely used dynamic covalent bonds are disulfide, hydrazone, borate esters, imines, and thioesters. The boric acid ester bond is a dynamic covalent bond formed by the coupling of boric acid with alcohols or phenols. Borate esters are unstable and are easily hydrolyzed and exchanged in the presence of o-diol and catechol (Wilson et al., 2014). Phenylboric acid (PBA) is a Lewis acid, which can form reversible borate esters with *cis*-diols such as carbohydrates and ribonucleotides (Chen et al., 2012; Kim et al., 2016b). Tumor-targeting nanoparticles mediated by PBA represent an attractive strategy for enhancing siRNA transport and treatment of metastatic cancer. However, the non-specific binding to various biofilms containing a *cis*-glycol structure limits the potential application of a drug delivery system. Fan et al. (2017a) constructed an siRNA nanodelivery system with dual functions of pH-responsiveness and tumor-targeting stemming from the reversible dynamic covalent borate bond formed by PBA and Cat based on catechol-modified polyethylene glycol (PEG-Cat) and PBA terminal polyethyleneimine (PEI-PBA), which can be used in the effective treatment of primary and metastatic tumors. Zhou et al. (2018) proposed a new reversible-covalent crosslinking strategy and prepared adenosine triphosphate (ATP)-grafted hydroxyapatite (HA) (HA-ATP) and PBA-modified PEI (PEI-PBA) to couple HA-PEI to borate bonds. By comparing the two siRNA delivery systems of physical adsorption and reversible-covalent crosslinking, the reversible-covalent crosslinking strategy is expected to turn on/off the connection between the extracellular stable state and intracellular unstable state, which can stimulate the disassembly of polymers in tumor cells and significantly improve the transport of siRNA (Zhou et al., 2018). Compared with PBA, boric acid polymers are more often used to construct reversible-covalent borate ester bonds. Bortezomib (BTZ) is an effective and specific proteasome inhibitor for cancer treatment (Wang et al., 2015). Su et al. (2011) coupled BTZ with a polymer carrier containing catechol through a reversible dynamic covalent borate ester bond and then synthesized a new type of cell-targeted and pH-sensitive polymer carrier by modifying biotin-targeted ligands that are selectively ingested by cancer cells through the mechanism mediated by cell surface receptors, thus delivering the anticancer drug BTZ to cancer cells. However, the activity of BTZ against solid tumors is lower; systemic use will lead to a higher risk of adverse reactions, and BTZ resistance has been observed. Wang et al. (2015) reported a pH-responsive polymer based on a polyamide dendrimer, which is functionalized by the catechol group inside the dendrimer and achieves the “off-on” release of BTZ in the slightly acidic tumor microenvironment through catechol–borate dynamic covalent bonding interactions with BTZ, allowing the therapeutic effect of BTZ in cancer treatment to be maintained while reducing its adverse reactions. Recently, in view of the factors that limit the clinical application of BTZ, such as low water solubility, instability, a non-specific distribution, and poor permeability to tumors, Zhao et al. (2019a) designed a new strategy without any organic synthesis and modification. Based on the dynamic and reasonable supramolecular self-assembly of BTZ, TA, and poloxamer (F68), a new high-load, high-stability, and controlled-release nanosheet was designed. Among them, the borate ester bond has unique room temperature dynamic reversibility and a multistimulus response, which can be used as a connection. It can not only improve the efficiency of drug loading but also reduce premature leakage. This study paves a possible way to solve the dilemma between functionality and medicine, as shown in Figure 8 (Zhao et al., 2019a). At present, some functional systems use a single type of dynamic covalent bond, but most have multiple interactions including dynamic covalent and non-covalent interactions. The application of the dynamic covalent bond in life science will focus on the coupling activation and inactivation of chemical disruptors, which may be identified by *in situ* selection (Wilson et al., 2014).

### Mixed Interaction Assembly of TA

**Figure 9:**
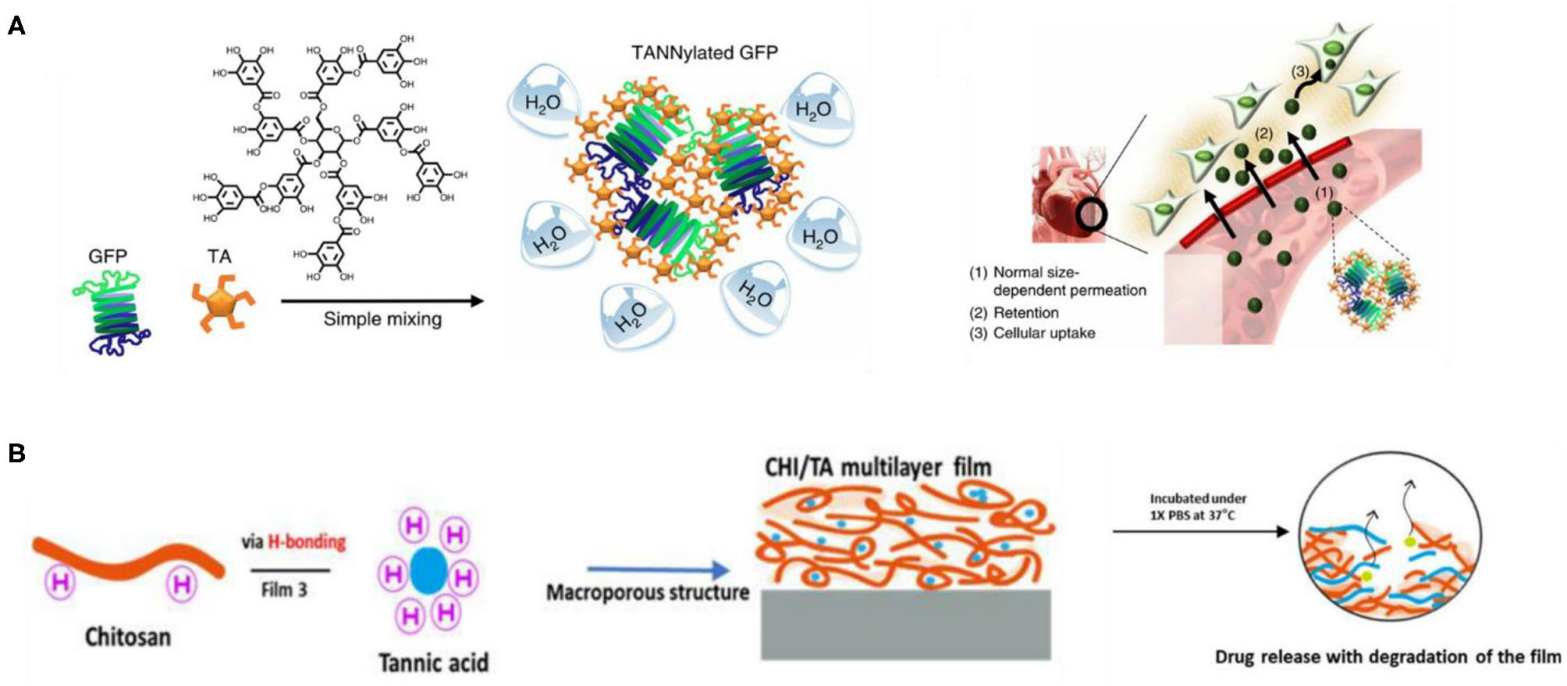
Mixed interaction assembly and application of tannic acid. **(A)** Schematic of the preparation of nanocomplexes of TANNylated GFP resulting in heart-targeting function (Shin et al., 2018). **(B)** CHI/TA film driven by electrostatic interactions and hydrogen bonds used as a sustained drug release carrier (Sun et al., 2020).

Despite some limitations in the stability and material selection of the supramolecular assembly process based on a single interaction, the cooperative assembly of multiple interactions can solve the above problems. The supramolecular assembly of TA with multiple interactions is carried out through dynamic covalent and non-covalent interactions such as static electricity, hydrogen bonding, hydrophobicity, and coordination, which are very common in TA and protein. Shin et al. (2018) found that TA can form multiple hydrogen bonds and hydrophobic interactions with many proteins, which is called TANNylation, and confirmed that systematic injection of TANNylated therapeutic proteins, peptides, or viruses may enhance the treatment of heart disease, as shown in Figure 9A. Bai et al. (2019) introduced TA into SF by electrostatic interactions and hydrogen bonding, and they developed a silk-based sealant with good moisture and instant hemostatic properties, which can be used as a promising surgical sealant for seamless sealing of ruptured tissues in wet and dynamic biological environments. Wang X. et al. (2018) prepared polyphenol–poloxamer self-assembled supramolecular nanoparticles (PPNPs), which have good biocompatibility due to multivalent hydrogen bonding between TA and PluronicF-127 combined with hydrophobic interactions on the poly(propylene oxide) chain, so PPNPs provide a good bimodal contrast agent for tumor imaging *in vivo*. Sun et al. (2020) demonstrated the possibility of developing a rapidly degradable chitosan-based multilayer film for controlled drug release and found that TA and DOX showed sustained drug release through electrostatic, hydrogen bond and hydrophobic interactions, as shown in Figure 9B. TA interacts strongly with proline-rich proteins, including salivary proteins, collagen, and gelatin. The PEI–gelatin–TA deposition method proposed by Ringwald and Ball (2016) can be used to prepare bioactive TA as antibacterial or antioxidant composite film materials conveniently and repeatedly. As a positively charged globular protein, lysozyme (Lys) can strongly bind to TA through hydrophobic interaction, hydrogen bonding interaction, and electrostatic interaction. Yang et al. (2019a) carried out a further study on the assembly of TA and gelatin. By taking advantage of the antioxidant properties of TA and the biocompatibility of gelatin, the TA/gelatin multilayers prepared by the LbL method showed good antioxidant properties, which are expected to improve the effect and success rate of implants. In addition, Yang et al. (2019b) used LbL to assemble and construct a multifunctional membrane (TA/Lys) with the characteristics of good oxidation resistance, rapid initial cell adhesion, enhanced osteogenesis, and broad-spectrum antibacterial, which is expected to be used in implant coating. It is worth noting that acidic lysozyme can also be assembled with graphene oxide (GO) by electrostatic, hydrophobic, π-π stacking, and van der Waals interaction. Li et al. (2019b) prepared a 10-nm ultrathin (GO/Lys)_8_ layer by LbL technology, which has a significant bacteriostatic effect on Gram-positive *Staphylococcus aureus* and Gram-negative *Escherichia coli* and can enhance the efficiency of osteogenic differentiation, so it has potential application in bone implant coating. The supramolecular assembly of TA based on multiple interactions has better properties than supramolecular materials assembled by a single interaction and attracts more research interest.

## Medical Applications of TA Self-Assembly

### Medical Application of Polyphenols

Polyphenols, including TA, EGCG, and catechin, are hydroxyl-rich molecules extracted from plants (Wang X. et al., 2018). The unique structure of polyphenol hydroxyl groups not only provides non-covalent bonds but also introduces dynamic covalent bonds. They have a wide range of biological characteristics, such as anti-inflammatory, anticancer, antibacterial, and antioxidation effects, resulting in their wide use in the field of biomedicine (Galante et al., 2019; Song et al., 2019). TA, as a kind of polyphenolic organic compound, has some structural properties and biological activity, and it can be used as a building unit for supramolecular assembly to obtain nanomaterials with biological functions.

EGCG, the most abundant and active polyphenol of green tea (Sundaramurthy et al., 2014), has strong binding affinity with some polymers via non-covalent bond interactions (Guillerm et al., 2012). Ren et al. (2020) used the coordination of metal–polyphenols to prepare a high-loading-capacity organic therapeutic nanodrug (PTCG-NPs) by using EGCG, Pt-OH, and PEG-b-PPOH as building blocks. The powerful metal polyphenol synergistic effect endowed PTCG-NPs with excellent stability in a physiological environment to achieve effective drug release, while activated cisplatin helped improve the level of hydrogen peroxide in cells. Through a cascade reaction, PTCG-NPs specifically opened the gates of cancer cells, releasing oxygen species and anticancer drugs to achieve the combination of chemo/chemodynamic treatment. By using Gd-doped PTCG-NPs, the imaging function was successfully introduced to monitor drug release and release behavior, providing nanodrug diagnostic ability. The translational potential of PTCG-NPs was discovered in *in vivo* studies, because they had excellent biocompatibility and synergistic antitumor effects (Ren et al., 2020). Compared with most traditional drug delivery systems, the hybrid technology of metal and polyphenol ligand improves the anticancer effect of nanodrugs, avoids the systemic toxicity of platinum drugs, and enriches the therapeutic function of nanodrugs. Their pioneering work not only provided a new strategy for the development of nanotherapeutic drugs but also expanded the application in the field of cancer treatment (Mathivanan et al., 2019). For the challenging problem of targeted therapy of metastatic melanoma, the assembly of EGCG and metal shows good results. Using green tea polyphenols, EGCG, and lanthanide metal ions (Sm^3+^) as building blocks, Li et al. (2019a) designed self-assembled Sm-III-EGCG nanocomplexes with synergistic antitumor properties. The systemic toxic effects of these nanocomposites on healthy cells are negligible, but the activity of melanoma cells is significantly reduced by effectively regulating their metabolic pathway. Green tea–based self-assembled nanocomposites have all the key features of promising clinical candidates and can address the challenges associated with the treatment of advanced metastatic melanoma (Li et al., 2019a).

BTZ is a first-class inhibitor of boric acid proteasome for cancer treatment. However, because of its complexation with dietary polyphenols, its therapeutic effect is severely inhibited. Inspired by this dynamic covalent chemistry, Wang et al. (2018b) proposed a polyphenol-mediated BTZ assembly strategy for a new type of supramolecular nanodrug for cancer treatment, which transformed natural polyphenols from enemies to friends. First, BTZ can simply combine with natural polyphenols containing catechol through borate bonds, and then it can conveniently form dynamic amphiphilic drugs, which possess pH-dependent assembly/disassembly behavior under special physiological conditions. Ferric ion was also incorporated into the supramolecular system via metal–phenolic coordination interactions to both introduce bioimaging functions and facilitate stability of the supramolecular nanomedicines. Second, Fe(III) ions were introduced into the supramolecular system through metal–phenol coordination, which not only endowed the biological imaging function but also promoted the stability of supramolecular nanodrugs. In addition, they chose to use four natural polyphenols with good biocompatibility and bioavailability, including catechin, EGCG, procyanidin, and TA, to confirm the versatility and modularity of the material design strategy. This unique theoretical system of supramolecular design has many advantages, such as high and precise control of drug loading, excellent biodegradation and biocompatibility, ease of operation and setup, and no requirement for presynthesis. The acquired supramolecular nanodrugs have a variety of biological functions, adjustable shapes and sizes, and limited side effects and can be used for a variety of diseases. Their work not only achieved a small drug assembly strategy based on natural polyphenols, which enables supramolecular nanodrugs to effectively release and control BTZ at targeted tumor sites, but also was very effective in treating cancer through significantly inducing tumor cell apoptosis and inhibiting tumor growth. In addition, their work promoted the further development of more kinds of natural polyphenol nanodrugs (Wang et al., 2018b).

### Advantages of TA Self-Assembly in Medical Applications

Because TA has a variety of biological activities, self-assembled supramolecular nanomaterials based on TA have unique advantages in the field of biomedicine. First, TA interacts with biomolecules and metal ions in bacteria to increase the cell membrane permeability, destroy the cell membrane stability, and change the protein-to-lipid ratio (Chung et al., 1998a), so that the assembled materials have good antibacterial properties. The phenol group in TA has good antioxidant activity, in which ROS can be eliminated by converting the phenol to a quinone group to consume ROS under oxidative stress, providing the assembled material with good antioxidant activity (Liu et al., 2018). TA combines with calcium ions, one of the six most important metal ions in the process of osteogenesis, so that the assembly material has a strong osteogenic effect (Xu et al., 2018). TA, which is easily metabolized by hydrolysis of the ester bond, can be biodegraded to gallic acid and glucose without cytotoxicity to the surface (Onat et al., 2018). Therefore, it provides the assembly material with good biocompatibility and safety. TA can form precipitable complexes with proteins in a non-specific manner (Franz et al., 2011), endowing the assembly materials with hemostatic properties. TA can inhibit membrane lipid peroxidation (Mathivanan et al., 2019), resulting in assembly materials with antiviral and antitumor activity. In addition, TA endows assembly materials with medical properties such as immunomodulatory effects (Chung et al., 1998b) and targeting of heart tissue (Shin et al., 2018), so self-assembled nanomaterials based on TA have attracted attention in medical applications.

In particular, the antioxidant activity of TA itself makes it a green natural reducing agent, which can reduce many kinds of metal ions such as Au^+^, Ag^+^, Fe^3+^, and Pd^2+^, and the use of natural plant polyphenol TA as a reducing agent is in line with the principles of sustainable and eco-friendly development. Fang et al. (2018a) synthesized AuNPs@TA core-shell nanocomposites using TA as the reducing agent, and it can be utilized as a sensor for Fe^3+^ and H_2_O_2_. Wang et al. developed a strategy for the preparation of antibacterial nanoparticle magnetic nanocomposites by alternately depositing Lys and TA. The reducibility of TA can reduce Ag^+^ to Ag nanoparticles *in situ*, which has a synergistic antibacterial effect with TA. Because TA can be oxidized to quinone by itself to achieve the purpose of reducing Ag^+^, the deposition of Ag nanoparticles does not require the addition of an additional reducing agent. Antibacterial experiments have shown that this nanocomposite has a good inhibitory effect on Gram-negative *E. coli* and Gram-positive *S. aureus*. In addition, because of the excellent magnetic response properties of IONPs (Fe_3_O_4_ nanoparticles), they are conducive to the recycling of nanocomposites, so this nanocomposite is an ideal material with green environmental protection and good antibacterial properties (Wang X. et al., 2017). In addition to metal ions, TA can reduce GO and supply material functionality. Lei et al. (2011) prepared TA-RGO (reduced GO) materials through the reduction of GO by TA and surface functional modification of GO. TA-RGO materials have good dispersibility in water and organic solvents. Therefore, we can consider the preparation of materials and functional polymers into composites. Kim et al. (2016a) prepared a polyamide reverse osmosis nanocomposite membrane with a TA-RGO active layer by polymerizing triformyl chloride, m-phenylenediamine, and TA-RGO on a polysulfone carrier. The experimental result showed that the polyamide film embedded in TA-RGO had better antibacterial properties than the polyamide film embedded in TA or GO alone.

Of course, as a biomaterial, TA has some limitations. For example, the ability of TA to inactivate protein (Ninan et al., 2016; Wang et al., 2016) and change the structure and stability of the cell membrane may harm human cells. Second, because of the chelating effect of TA on metal ions, overuse may cause diseases such as iron deficiency anemia and calcium deficiency. In addition, the use of TA on large wounds may lead to absorptive poisoning (Song et al., 2019). Supramolecular assembly can functionalize TA or assemble TA into nanostructured composites through assembly regulation, and the concentration of assembly materials can be greatly reduced by LbL assembly, thus reducing the limitations of TA materials.

### Self-Assembly System and Application of TA

Supramolecular self-assembly using TA as building blocks can generate different materials, such as hydrogels, nanoparticles/microparticles, hollow capsules, and coating films, resulting in enormous potential medical applications including drug delivery, tumor diagnosis and treatment, bone tissue engineering, biofunctional membrane materials, and the treatment of certain diseases.

### Hydrogels

**Figure 10:**
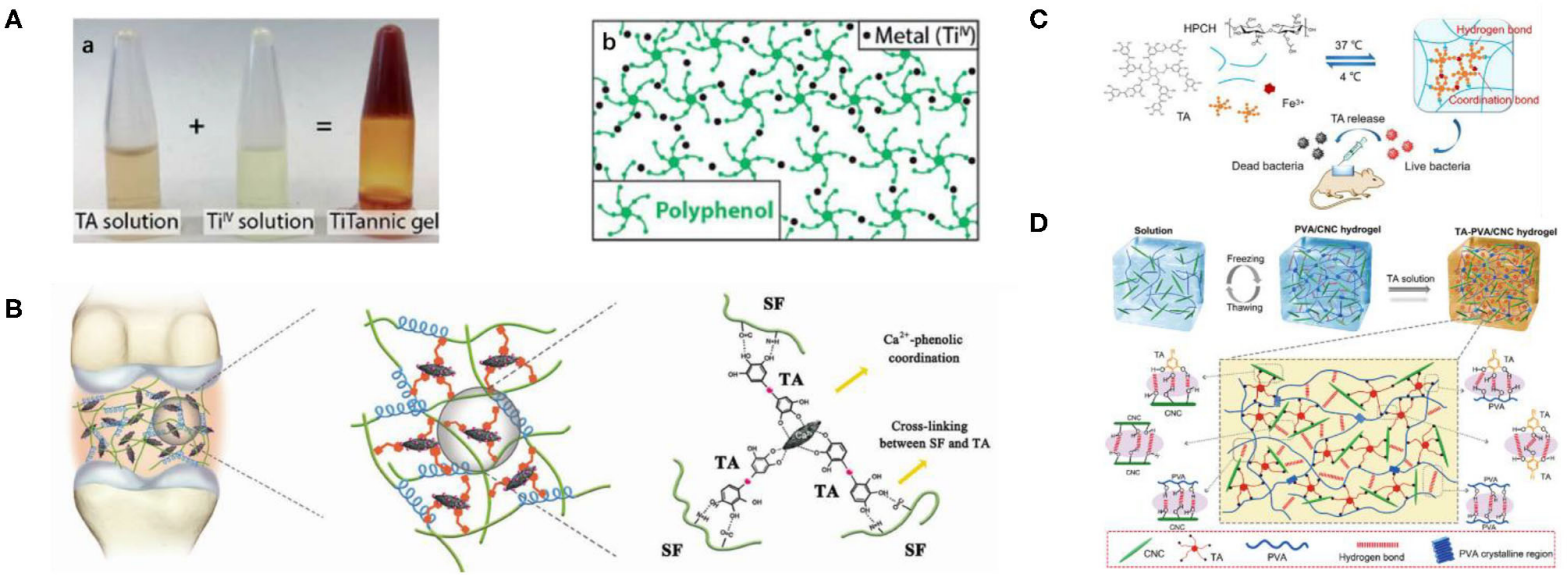
Hydrogel based on TA and its application. **(A)** Supramolecular gelation based on TA and metal. (a) The formation process of the TA-Ti hydrogel. (b) Schematic illustration of the gel stabilized by the coordination between the metal ion and polyphenol (Bjornmalm et al., 2019). **(B)** The proposed mechanism schematic for the high toughness of SF@TA@HA (Bai et al., 2020). **(C)** The synthesis and possible schematic of the HPC H/TA/Fe hydrogel (Ma et al., 2020). **(D)** The fabrication and possible mechanism of TA–PVA/CNC hydrogels (Lin et al., 2020).

Natural hydrogels, such as extracellular matrix or mucus, have the ability to regulate a series of biological properties of macromolecules and cells (Crouzier et al., 2012). Therefore, biomimetic supramolecular hydrogels with biological functions have been studied. Supramolecular hydrogel is a three-dimensional polymer network with high water content formed by connecting small molecules in a non-covalent way, which has many properties, such as biocompatibility (Calo and Khutoryanskiy, 2015), excellent cell adhesion (Jen et al., 1996), biodegradability (Kamath and Park, 1993), and ease of molecular transport (Hoffman, 2012), leading to a variety of biomedical applications. TA, as a kind of economical and effective natural polyphenol compound, has a polyphenol-arm structure, which can grasp the polymer chain through a hydrogen bond, ion bond, and coordination bond. Synthetic hydrogels were first discovered by Wichterle and Lim (1960). Self-assembled hydrogels based on TA first appeared in 2015. DNA-TA film hydrogels based on hydrogen bonds were synthesized by Shin et al. (2015) via LbL assembly technology, who found that DNA/TA hybrid hydrogels have biodegradability, ductility, tissue adhesion, and hemostatic abilities. Fan et al. (2017b) developed a new strategy for crosslinking available polymers with -N- or -O- units into supramolecular hydrogels via acid interactions based on TA, a classical multifunctional material with rapid self-healing, mechanical durability, pH-responsive, and free radical–scavenging activities. The construction of supramolecular hydrogels consisted of two programs. (1) TA assembled the dynamic part via a hydrogen bond or ionic bond with a polymer chain. (2) These polymer chains were cross-connected to networks by coordination bonds with Fe(III). By adjusting the weight ratio of polymer/TA and TA/Fe^3+^, these two interactions could be well-balanced, which is the key to constructing supramolecular hydrogels. In addition, the mechanical properties of the obtained hydrogels are regulated by the interaction balance. When the optimal proportion of TA/Fe(III) is maintained at 3:5, the component density—that is, the ratio of TA/polymer—increases, the moduli are enhanced, and the mechanical strength can be adjusted from 10 Pa to 10 kPa. Their work confirmed that TA provides a simple and feasible approach for the construction of multifunctional hydrogels, as a multifunctional and versatile catechol group modifier, which has advantages including simple processing, low cost, and a large preparation scale (Fan et al., 2017b). Considering the hydrogels prepared *in vivo* with features including minimal invasiveness, ease of use, and high translational potential, Bjornmalm et al. (2019) successfully prepared a metal–phenol supramolecular hydrogel by TA and Ti(IV) *in vivo*. Subsequently, they provided an essential characterization of the prepared hydrogel by optical and electron microscopy, Raman spectroscopy, and rheology and evaluated the permeability and porosity of the hydrogels by glucose permeability and particle tracking analysis, respectively. The results showed that the TA-Ti(IV) hydrogel not only was stable and well-tolerated but also triggered a faint and persistent foreign body reaction. In addition, the biological distribution of titanium was studied by mass spectrometry. Although 14 weeks had passed, titanium in the samples basically remained at the basic level, indicating that the accumulation of titanium in the distal tissue was very low and even negligible. Compared with the poloxamer hydrogel, the prepared TA-Ti(IV) hydrogel showed a more sustained release (from <1 days to >10 days) when the corticosteroid dexamethasone was used clinically. Apparently, their research provided a solid foundation for further development of the titanium-containing hydrogel in biomedical fields, such as drug delivery and regenerative medicine, as shown in Figure 10A (Bjornmalm et al., 2019). Although hydrogel-based bone adhesives are expected to revolutionize the clinical treatment of bone repair, they still have serious deficiencies, such as inappropriate mechanical strength, cytotoxicity, and poor fixation performance. Based on the unique role of gum molecules in bone strength and flexural strength, Bai et al. (2020) introduced natural polyphenol as a powerful synthetic tool for the preparation of high-performance biocompatible bone adhesives, which can avoid the defects of traditional hydrogels, enhance water-resistant fixation, and guide bone regeneration. They used TA, SF, and HA to prepare SF@TA@HA hydrogel by self-assembly, which has good biocompatibility, controllable biodegradation, strong wet adhesion ability, and broad-spectrum antibacterial activity. The synergistic effect between the strong affinity of SF and TA and the coordination bond of TA and HA resulted in excellent immobilization properties of the hydrogel. Their work proposed a biomimetic bone adhesive that can provide stable fracture fixation and accelerate bone regeneration during bone reconstruction, as shown in Figure 10B. In clinical applications, it is very important but challenging to prevent scarring and accelerate wound healing, especially infection. Ma et al. (2020) prepared a new thermosensitive and pH-responsive composite hydrogel (HPCH/TA/Fe) based on the simple assembly of HPCH, TA and Fe^3+^. As a structural unit, TA can not only dynamically associate with HPCH and Fe^3+^ via non-covalent bonding but also be used as a pH-dependent and sustained-release antibacterial agent. It was found that the prepared hydrogel had good biocompatibility and long-lasting antibacterial activity. It could also effectively inhibit bacterial infection and accelerate wound healing without scarring. The hydrogel synthesized by their group is expected to have good application prospects in the treatment of infected wounds, as shown in Figure 10C. Inspired by natural biological protein materials, Lin et al. (2020) used TA as a molecular coupling bridge between cellulose nanocrystal (CNC) and the polyvinyl alcohol (PVA) chain to prepare a biobased advanced physical hydrogel through strong hydrogen bonds. These biomimetic hydrogels have remarkable toughness, ultrahigh strength, high elongation, good self-recovery, extensive adhesion, and good antibacterial properties, resulting in broad application prospects in the field of tissue engineering and biomedicine, as shown in Figure 10D. In recent years, dynamic hydrogels based on macrocyclic host–guest interaction have attracted the attention of researchers. Different supramolecular hosts are introduced into hydrogels, and dynamic hydrogels with different properties can be obtained by physical or chemical crosslinking. These hydrogels have great application potential in the fields of biomedical materials, self-repairing materials, and intelligent materials. However, the formation mechanism of dynamic hydrogel still must be further studied, and as a biomedical application, hydrogel needs more extensive research in biocompatibility and cytotoxicity testing (Xiao et al., 2019b). In addition, the poor mechanical stability (Hoffman, 2012) and rapid drug release of hydrogels (Brandl et al., 2010) limit their development.

### Nanoparticles/Microparticles

**Figure 11:**
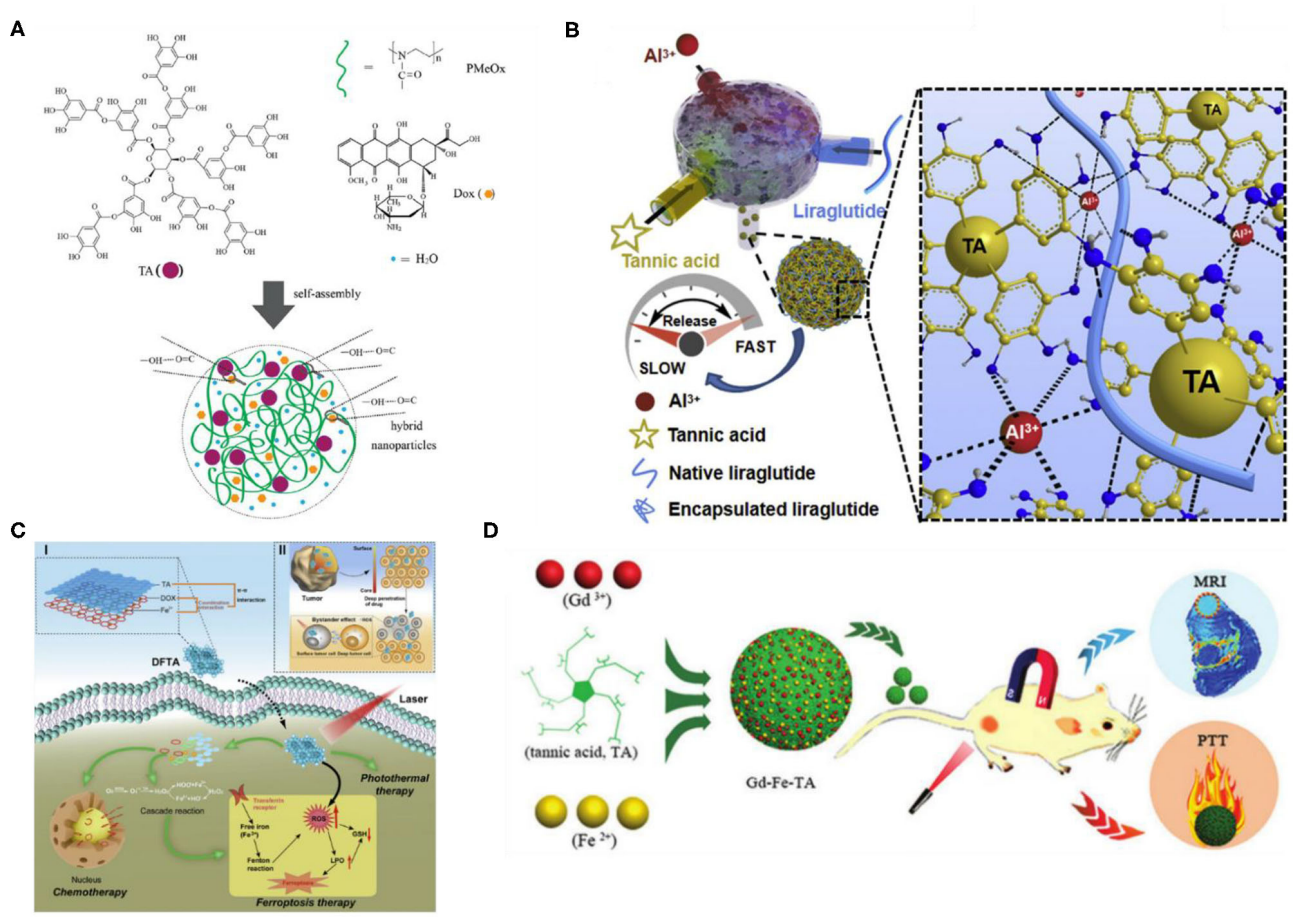
Nano/microparticles based on TA and its application. **(A)** Chemical structures of TA, PMeOx, and Dox and a schematic of nanoparticles based on them (Liu et al., 2020). **(B)** Schematic illustration of the ternary nanoparticle assembly based on TA and Al^3+^ (He et al., 2020). **(C)** The assembly of DFTA driven by π-π interactions and coordination reactions, as well as triple combination therapy mechanisms of DFTA after targeted uptake by tumor cells (Xiong et al., 2019). **(D)** The synthesis and applications of polymer nanoparticles based on bimetal–TA (Qin et al., 2020).

Polymer nanoparticles/microparticles based on non-covalent interaction are a kind of soft material that can be used as effective delivery systems with properties of a stimulus response, self-healing, and controlled release, and they are very attractive in biomedical and related applications (Wang et al., 2018a). Nanoparticles/microparticles based on hydrogen bonding have been widely studied as a delivery system in recent years. By using the intermolecular hydrogen bond and electrostatic interaction between neutral poly(2-methyl-2-oxazoline) (PMeOx), TA, and adriamycin hydrochloride (Dox), Liu et al. (2020) developed a direct coassembly strategy for the preparation of PMeOx-TA-Dox nanoparticles. The prepared spherical nanoparticles possessed advantages of good water dispersion, stability, and biocompatibility. In addition, PMeOx-TA-Dox nanoparticles had pH-dependent drug loading and release behavior and cell uptake characteristics, as Dox and PMeOx-TA can be separated rapidly in fetal serum, which can promote Dox release and entry into the nucleus. Their work was expected to reveal a new therapeutic drug carrier, as shown in Figure 11A (Liu et al., 2020). In addition, researchers have studied nanoparticles/microparticles based on coordination. He et al. (2020) developed Lira/TA/Al^3+^ternary nanoparticles based on the formation of a hydrogen bond between Lilaru peptide and TA and the stability of the complexation of TA with Al^3+^, which can be used to treat T_2_D by frequent administration of lira peptide, to overcome the shortcomings of low bioavailability and lira with a short half-life. The high stability and controllable size of nanoparticles are mainly determined by the hydrogen bond between lira and TA as the main driving force and the coordination of Al^3+^ and TA as an additional crosslinking effect. This nanoparticle system shows better therapeutic potential because of its long-term blood glucose control, improved cardiovascular function, and reduced tissue toxicity of multiple organs, as shown in Figure 11B (He et al., 2020). Xiong et al. (2019) used doxorubicin as a chemotherapeutic drug, ferric trichloride (FeCl_3_) as an iron prolapse inducer, and TA as an intracellular superoxide dismutase–like reaction activator to construct a drug-organic-inorganic self-assembly nanosystem (DFTA) for the combined treatment of ER^+^ breast cancer. The assembled nanosystem is based on coordination and π-π interactions. The authors found that the oxidative stress disorders were antagonized by intracellular oxidation–reduction cascade reactions, ferroptosis, and PT, thus endowing the DFTA^+^ laser system with the effect of accurately attacking ER^+^ breast cancer, which has obvious antitumor effects and low toxicity. Their highly effective nanosystem based on chemotherapy, iron decline, and platinum is expected to be a new approach for the effective treatment of ER^+^ breast cancer, as shown in Figure 11C. Qin et al. (2020) used gadolinium nitrate and ferrous sulfate as metal sources and plant polyphenols (TA) as organic ligands to synthesize bimetallic–phenolic coordination polymer nanoparticles by the metal–catechol coordination assembly process, which can not only effectively enhance tumor signals as contrast agents but also effectively inhibit tumor growth by photothermotherapy. This work provides a new idea for the synthesis of multifunctional coordination polymer nanoparticles and expands their potential applications in the field of acoustics, as shown in Figure 11D. Although the preparation of nanoparticles has been widely studied in recent years, the low efficiency of preparation technology, tedious steps, and other factors limit their wider application, so how to obtain stable nanoparticles more simply and efficiently may be a future research direction.

### Hollow Capsules

**Figure 12:**
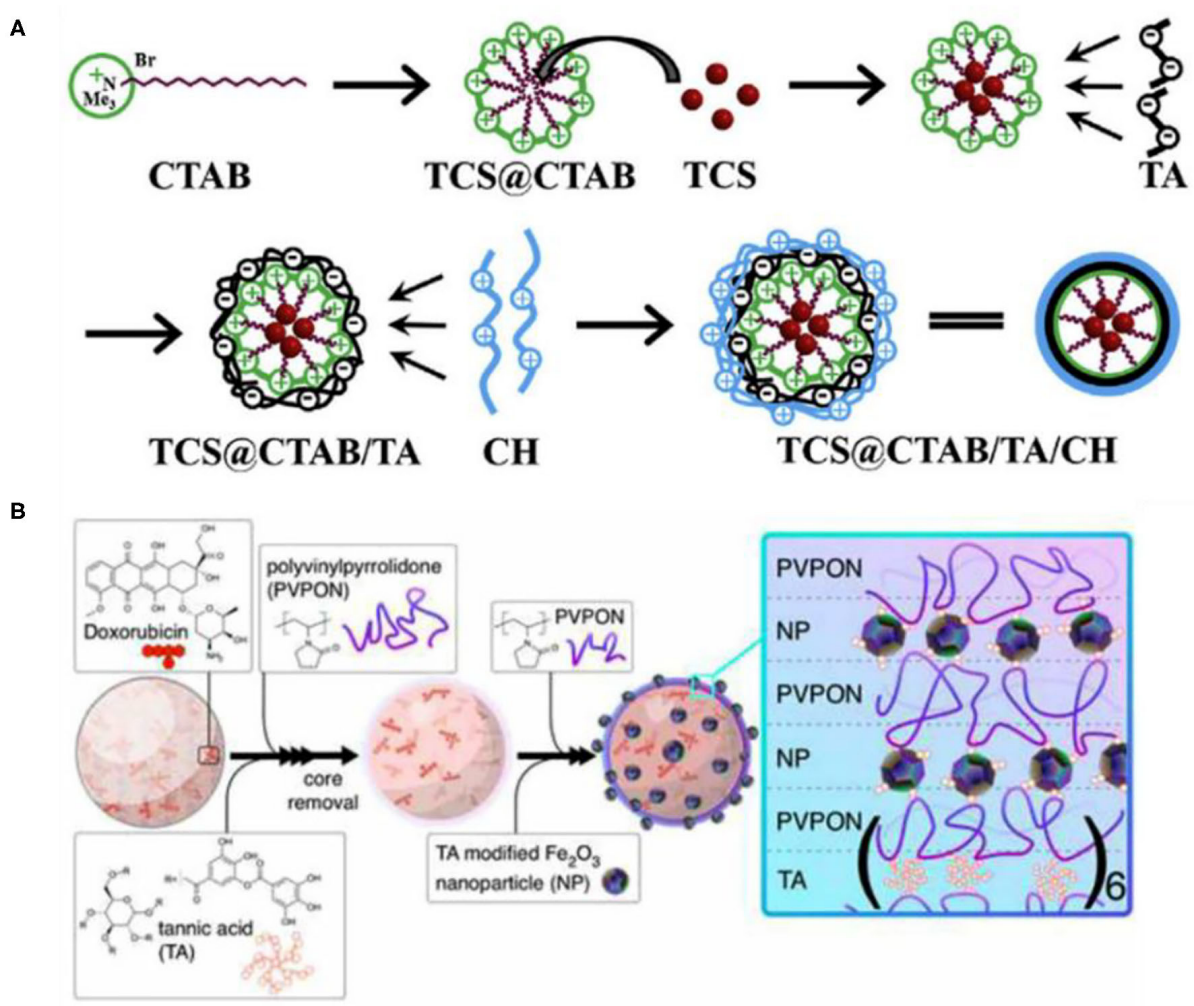
Hollow capsules based on TA and their application. **(A)** The preparation of nanocapsules via electrostatic assembly (Cai et al., 2019). **(B)** The assembly of hollow (TA/PVPON)_n_ capsules (n denotes the number of bilayers) on doxorubicin-loaded porous 3-μm SiO_2_ microparticles. The final shell composition [(TA/PVPON)_6_(Fe_2_O_3_/PVPON)_2_] was obtained by depositing Fe_2_O_3_ nanoparticles (NPs) modified by TA in alternating layers with PVPON (Alford et al., 2018).

Hollow capsules fabricated via LbL assembly of polymers comprise an ultrathin permeable shell (<50 nm) that participates in various types of interactions (Mathivanan et al., 2019; Wang et al., 2020) and microsized or nanosized cavities that deposit drugs or active molecules. The shells are able to trigger the release of loaded molecules to protect the load from external stress and thus extend the time of release. The initial related research has mainly focused on the assembly of metal coordination capsules, and the engineering of metal-specific functions for capsule design is rarely explored. Guo et al. (2014) reported that a multifunctional capsule assembled by an MPN is prepared by a phenolic ligand (TA) and a series of metals. The film thickness, disassembly characteristics, and fluorescence behavior of MPN capsules can be controlled by coordination metals. In addition, the functional properties of MPN capsules are customized for drug delivery, positron emission tomography, magnetic resonance imaging (MRI), and catalysis (Guo et al., 2014). An excellent feature of LbL capsules is that they are capable of accurately controlling the transport and release of drug or biologically active molecules by inherent responsiveness against environmental and external triggers (Mathivanan et al., 2019). The risk of instability in the chemical and physical properties of capsules can be minimized by changing the layer number and polymer species in the shell (Alford et al., 2018). The unique properties of hollow capsules have resulted in their wide attention in various fields, such as nanoreactors, sensors for nanomedicine, and drug delivery for disease diagnosis and treatment (Wang P. et al., 2018). Mathivanan et al. (2019) prepared a capsule and coating (TA/PnPropOx) based on the LbL assembly technology of TA and PnPropOx, where natural polyphenol TA was used for the hydrogen-bond donor. They found that the temperature and pH of the assembly process had an apparent influence on the physical properties of the capsule, including growth, morphology, and stability. The capsule also showed good stability at pH 2–9, which might be attributed to the dehydration of TA/PnPropOx during LbL assembly. Because the side chain controls the hydrophilic and hydrophobic balance in the capsule, it is necessary to study the influence of the two-position side chain in poly(2-oxazoline) on the encapsulation performance and permeability of the capsule in the future. In conclusion, the capsules they prepared showed considerable potential for drug release (Mathivanan et al., 2019). Cai et al. (2019) first prepared novel nanocapsules (TCS@CTAB/TA/CH) using an electrostatic LbL assembly method with the alternative imine bond as an assembly driving force, which can be used for drug introduction and controlled release. When the pH changed from 8 to 4, the release efficiency of TCS increased by 61.8%, which was mainly because the TA/CH could be used as a pH bacterial-triggered valve to control TCS release, indicating that the prepared nanocapsules had good pH-responsive activity. Subsequently, the nanocapsules and dextran aldehyde (DA) could be assembled into pH-responsive (DA-TCS@CTAB/TA/CH)_n_ multifilms with good antibacterial and adhesion resistance through the imine bond. The controllable release function of the films positions them as a new antibacterial material for surface modification of antibacterial materials, as shown in Figure 12A (Cai et al., 2019). Alford et al. (2018) introduced a novel 3-μm-diameter biocompatible microcapsule composed of TA and poly(N-vinylpyrrolidone) and 4-nm iron oxide nanoparticles via LbL deposition in 2018, which can be used as a contrast agent to achieve targeted drug delivery and real-time tracking by combining MRI and ultrasound-triggered drug release technology. The characteristics of these materials include a long cycle; active contrast; a customizable shape, size, and composition; and precise high payload delivery, which provide a safe and powerful platform as efficient contrast-enhanced imaging agents to facilitate real-time tracking and targeted delivery of encapsulated drugs, showing great value in biomedical imaging, as shown in Figure 12B (Alford et al., 2018). Future research on the supramolecular assembly of nanocapsules should focus on the following points: (1) to find assembly materials with reasonable rates and biosafety of biodegradation, (2) to improve the loading and release efficiency of hollow capsules, (3) to accurately achieve the release of hollow capsules at the determined site through environmental response or an external trigger, and (4) to achieve the multifunctional integration of hollow capsules for biomedical applications.

### Coating Films

**Figure 13:**
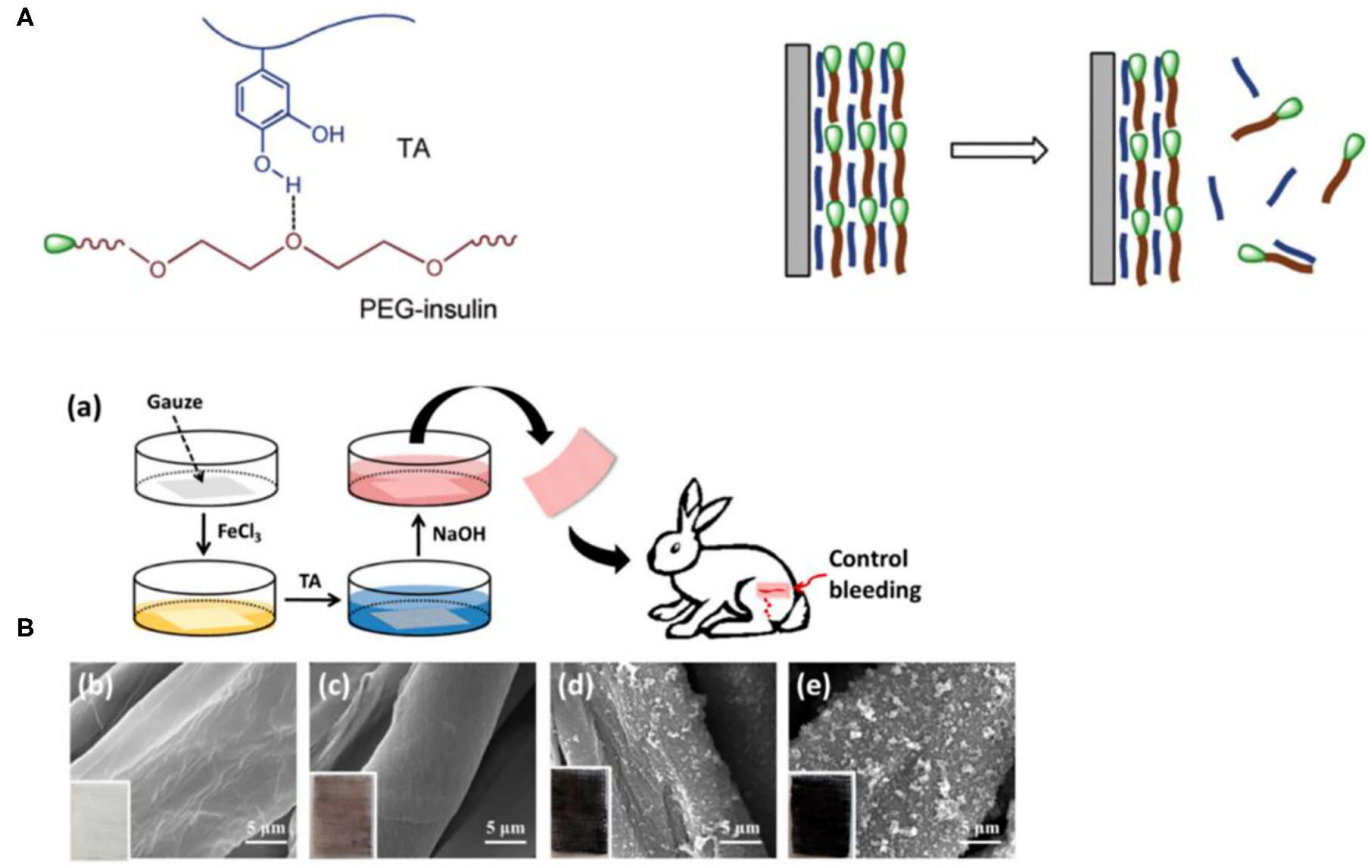
Coating films based on TA and their application. **(A)** The interaction of PEG–insulin and TA was driven by hydrogen bonding, and gradual disintegration of the PEG–insulin/TA LbL film led to the release of PEG–insulin (Wang et al., 2020). **(B)** (a) The preparation and application of the TA-coated gauze in bleeding control [SEM images of the gauze deposited with 0 (b), 1 (c), 3 (d), and 5 (e) TA deposition cycles] (Song et al., 2019).

Self-assembled coatings for surface modification and medical potential have become a hot research topic. The assembled coating has a good adjustable structure and reproducibility, so it is widely used in many medical fields, including dental implants and bone coatings. Ejima et al. (2013) first proposed synthesis of coordination complexes using natural polyphenol TA as an organic ligand and Fe(III) as an inorganic crosslinking agent, which can be used to prepare various films and particles on a series of substrates by one-step assembly. This coordination complex assembly based on metal–polyphenols is a milestone for the development of a simple and general strategy for thin film and particle engineering (Ejima et al., 2013). The one-step assembly of coordination complexes for versatile film and particle engineering not only gives assembly the advantages of a simple process, mild manufacturing conditions, varied materials, and low equipment cost but also allows the coating of a variety of substrates with different complex shapes and the precise control of coating thickness up to the nanometer scale. To achieve a persistent, flat, and stable insulin supply, a new drug carrier was designed. Wang et al. (2020) assembled PEG–insulin and TA into a film, which can release PEG–insulin into the medium through a reversible, dynamic hydrogen bond. In addition, the unique release mechanism of the films explained the unique release kinetics of PEG–insulin, which had zero-order kinetics. The composite films can help reduce fasting blood glucose level that are close to normal for an extended period. Of note, the thickness of the film is closely related to the reaction time. Their work was promising in the treatment of diabetes, as shown in Figure 13A (Wang et al., 2020). Zhu et al. (2019) constructed a novel multifunctional thin-film nanofibrous composite membrane based on TA and polyvinyl alcohol (PSBMA) on TFNC membrane surfaces by LbL assembly, expecting to achieve the filtration of dye and protein. The composite membranes had not only excellent blood compatibility, such as a great reduction of platelet adhesion, reduction of the hemolysis rate, and significant prolongation of the clotting time, but also good antibiological pollution performance, including lower bovine serum albumin (BSA) adsorption, better hydrophilicity, less *E. coli* and *S. aureus* bacterial attachment and higher water flux recovery. Their zwitterionic multilayers demonstrated broad application prospects in the fields of water treatment and blood contact biomedicine and provided a useful idea for the preparation of biocompatible coatings on various substrates (Zhu et al., 2019). By chelating TA and Fe^3+^ ions, Song et al. (2019) assembled the TA coating on medical gauze as a hemostatic dressing to solve the problem of potential absorption poisoning with large-area wound TA. The study found that the TA coating possessed excellent adsorption on proteins including BSA, immunoglobulin G, and fibrinogen. In addition, it had a good hemostatic performance in the process of animal wound coagulation due to erythrolysis and protein adsorption, especially the fibrinogen associated with blood clotting. Their coating with features, including economical, environmentally friendly and flexibility characteristics, demonstrated the potential to become a new type of hemostatic material in the medical field, as shown in Figure 13B (Song et al., 2019). Kumorek et al. (2020) prepared LbL films based on TA as cationic modifiers and pristine chitosan, which was modified with N-(2-hydroxypropyl)-3-trimethylammonium chloride. The films showed excellent antibacterial properties and an obvious pH dependence. They found that electrostatic interactions mainly drove the formation of the complex, while hydrogen bonding accompanied the process. The pH triggered the decomposable LbL membrane for use as a degradable coating, allowing the release of therapeutic drugs in biomedical applications and preventing bacterial adhesion (Kumorek et al., 2020). Because the preparation process of TA supramolecular assembled nanofilm coating is simple, the structure is controllable, and it can be coated on almost all substrates, endowing it with great prospects in bone tissue engineering and the functionalization of biomaterials. The antibacterial application of medical devices has great prospects, and the assembled film coating can be assembled by hydrogels, microparticles/nanoparticles and capsules, thus enriching the structure and function of the assembly materials.

Although various functional nanomaterials based on TA self-assembly have attracted increasing attention, some problems related to TA or the histidine complex should not be overlooked. For example, the practical use of antioxidants and chelating agents based on TA in the gastrointestinal tract is limited by the ability of tannins to bind to proteins in foods in non-specific ways, forming insoluble sediments and aggregates. TA has also been reported to inhibit the activity of digestive enzymes (Zhao et al., 2013). Similar to the self-assembled membrane of TA, on the one hand, the pH value has great prospects for the performance of membrane (Shutava et al., 2005; Zhou et al., 2013), because the dissociation of the phenolic hydroxyl group and direct breaking of the hydrogen bond cause the rapid destruction of the film in high pH solution. On the other hand, the stability of the membrane under physiological conditions has a negative impact on the reactivity of the capsule shell (Richert et al., 2004). In addition, when the polymer and TA are mixed in aqueous solution, hydrogels are often not obtained because of the dependence on hydrogen bonds or ionic interactions rather than covalent grafting but show homogeneous solutions or agglomerations. However, with the addition of Fe^3+^ and the pH adjustable coordination interaction between TA and Fe^3+^, an ideal hydrogel is obtained (Fan et al., 2017b).

## Conclusion and Future Perspectives

In the past decades, supramolecular assembly technology has been greatly enriched and developed, from traditional dip-coating assembly, spin-coating assembly, spray assembly, jet assembly, and electromagnetic assembly to the current lithography technology, three-dimensional printing and DPN (DPN is the technology of transferring alkyl mercaptan to a gold surface using an atomic force microscope cantilever tip), as well as dynamic film, coordination drive assembly and stereo complex assembly technology (Richardson et al., 2016). The regulation of supramolecular assembly is more accurate, which gives more functions to materials. Thus, supramolecular assembly based on TA has achieved great development in a short time. Although the existing supramolecular assembly technology is not applied in its entirety to the assembly of TA, the development of cutting-edge technologies such as lithography and DPN may push the supramolecular assembly of TA to a new level.

TA has special structural properties and biological activity. The polyhydroxyl structure of TA endows it with the ability to assemble supramolecular nanomaterials through a variety of non-covalent and covalent interactions. Additionally, the biocompatibility and antibacterial and antioxidant activities of TA allow supramolecular assembly materials to possess a variety of biomedical applications. The precise regulation of the structure by supramolecular assembly, combined with the structure and biological activity of TA, gives the supramolecular assembly nanomaterials of TA unique advantages in the fields of drug delivery, bone tissue engineering, and functional membrane coating, among others.

Although supramolecular assembly based on TA has many advantages, some challenges remain. From the dimension of the driving force of TA supramolecular assembly, the cationic polymer as a construction unit in assembly materials by electrostatic interactions may cause problems of cytotoxicity. Thus, the search for a low charge density, safe profile after biodegradation, and non-cytotoxic cationic polymers as construction units can solve the problem of cytotoxicity of electrostatic assembly. Although the assembly materials based on hydrogen bonding do not introduce cationic polymers, the assembled materials are unstable relative to electrostatic assembly due to the influence of pH, ionic strength, and other factors. However, the assembly process based on coordination is slow and can easily introduce impurities. In addition, other interactions, such as hydrophobic, charge transfer interaction, and host–guest interactions, as well as π-π stacking, are generally not used as the driving force of assembly alone but function together with other interactions to drive the assembly of TA supramolecular materials. Generally, supramolecular assembly with a single non-covalent interaction is relatively unstable, and the selection of assembly materials is few, so the development of mixed interaction assembly of TA may assemble more stable nanomaterials, and it may also be a good strategy to expand the variety of assembly materials. From the dimension of the TA assembly system, hydrogel supramolecules have limitations of poor mechanical stability (Hoffman, 2012) and rapid drug release (Brandl et al., 2010). The low efficiency and tedious steps of the preparation of microparticles/nanoparticles limit their wide application. For the hollow capsule system, how to improve the loading efficiency of the hollow capsule, accurately control the release, and achieve the multifunctional integration of hollow capsule biomedical devices remains as a challenge. In addition, the membrane coating system can be assembled by hydrogels, microparticles/nanoparticles and capsules to enrich the structure and function of the assembly materials, which may be an ideal strategy to change the TA assembly defects.

Supramolecular assembly materials of TA have been applied in the fields of drug delivery, tumor diagnosis and treatment, bone tissue engineering, biological functional membrane materials, and the treatment of certain diseases. It is believed that with the gradual deepening of the study of plant polyphenols and the development of supramolecular assembly technology, the application value of supramolecular nanomaterials based on TA will be better developed, and it will be more widely used in the field of medicine.

## Author Contributions

RL collected the literature involved in the review, wrote the draft of the review, and revised its format. XZh supplemented the content of the review. XC and LZ put forward suggestions for revision of the content of the review. XZa and YZ put forward constructive suggestions for review writing and revision. All authors contributed to the article and approved the submitted version.

## Conflict of Interest

The authors declare that the research was conducted in the absence of any commercial or financial relationships that could be construed as a potential conflict of interest.
